# Connective tissue disorders and cardiac arrhythmias: genetic pathways, mechanistic bridges, and clinical implications

**DOI:** 10.3389/fcvm.2026.1860012

**Published:** 2026-09-03

**Authors:** Luke Dreher, Hussein Abdul Nabi, Ramzi Ibrahim, Mahmoud Abdelnabi, Omar Baqal, Christopher N. Kanaan, Min Choon Tan, Linnea M. Baudhuin, Fadi E. Shamoun, Hicham Z. El Masry

**Affiliations:** 1 Department of Cardiovascular Medicine, Mayo Clinic Arizona, Phoenix, AZ, United States; 2 Department of Laboratory Medicine and Pathology, Mayo Clinic, Rochester, MN, United States

**Keywords:** cardiac arrhythmias, connective tissue disorders, extracellular matrix, fibrosis, mechanotransduction, mitral annular disjunction, mitral valve prolapse, TGF-β signaling

## Abstract

**Background:**

Connective tissue disorders (CTDs) such as Marfan syndrome, Loeys-Dietz syndrome, and Ehlers-Danlos syndromes are traditionally defined by vascular fragility and aortic complications. Increasing evidence, however, demonstrates that CTDs also predispose to atrial and ventricular arrhythmias, often independent of overt cardiomyopathy or severe valvular disease.

**Content:**

This review integrates emerging data on the arrhythmogenic substrates of CTDs. Four mechanistic bridges are emphasized: (1) diffuse myocardial fibrosis driven by dysregulated TGF-β signaling, (2) structural valve and annular abnormalities, particularly mitral valve prolapse and mitral annular disjunction, (3) altered mechanotransduction linking extracellular matrix instability to cytoskeletal and nuclear signaling pathways, and (4) downstream ion-channel and gap-junction remodeling as secondary amplifiers. We outline the genetic architecture underlying these processes, focusing on established CTD-implicated genes in the TGF-β/SMAD pathway, valve and annulus regulators, cytoskeletal and myocyte-coupling proteins, and extracellular matrix components, while incorporating emerging CTD modifiers that expand the spectrum of arrhythmia susceptibility.

**Summary:**

Recognition of CTDs as both vascular and electrical disorders reframes clinical management. Imaging markers such as mitral annular disjunction and diffuse fibrosis, together with targeted ambulatory rhythm monitoring and consideration of genetic context, may improve recognition of higher-risk phenotypes. Therapeutic strategies must balance arrhythmia control with procedural risk in fragile connective tissue substrates.

**Conclusions:**

By unifying genetic, mechanistic, and clinical insights, this review provides an integrated framework for understanding arrhythmias in CTDs. It underscores the need for prospective registries and genotype-phenotype studies to refine surveillance and prevention strategies.

## 1 Introduction

Connective tissue disorders (CTDs) such as Marfan syndrome (MFS), Loeys-Dietz syndrome (LDS), and the Ehlers-Danlos syndromes (EDS) have historically been studied primarily in the context of vascular complications, particularly aneurysm and dissection. However, emerging data highlight arrhythmias as an important but under-recognized source of morbidity and mortality in these conditions. Recent reports document atrial fibrillation, ventricular tachyarrhythmias, and conduction disease in CTDs, often independent of severe ventricular or valvular disease. This evolving evidence positions CTDs not only as vascular disorders but also as systemic substrates for electrical instability.

The most well-established arrhythmogenic phenotypes are mitral valve prolapse (MVP) and mitral annular disjunction (MAD), frequently observed in MFS and other CTDs. Studies demonstrate that MVP/MAD can produce basal left ventricular fibrosis, Purkinje-related ectopy, and malignant ventricular arrhythmias, even in the absence of severe mitral regurgitation (1, 2). Beyond valve geometry, multicenter studies of genetically characterized heritable thoracic aortic disease cohorts have reported atrial and ventricular arrhythmias among carriers of TGF-β pathway variants, implicating primary myocardial remodeling as part of the phenotype (3).

Several pathophysiologic mechanisms explain how CTD biology converges on arrhythmogenesis. First, excess TGF-β signaling and extracellular matrix (ECM) dysregulation drive diffuse interstitial fibrosis, a substrate for conduction heterogeneity and reentry (4, 5). Second, altered valvular and annular geometry (MVP/MAD) amplifies mechanical traction on papillary muscles and Purkinje fibers, producing focal fibrosis and promoting triggered activity. Third, abnormal mechanotransduction within cardiomyocytes links altered ECM stiffness to electrical remodeling, calcium mishandling, and arrhythmic vulnerability. These mechanisms converge on downstream processes also observed in inherited cardiomyopathies.

Despite growing recognition of arrhythmias in CTDs, existing literature remains fragmented: valve-focused studies highlight MVP and MAD, genetic reports emphasize myocardial disease, and aortic cohorts rarely incorporate rhythm outcomes. To date, no comprehensive review has unified CTD genetics, myocardial substrate biology, and arrhythmia phenotypes into a single framework. This review provides the first integrated genetic and mechanistic synthesis of arrhythmias in CTDs, highlighting how structural, fibrotic, and electrical processes intersect, and outlining implications for surveillance, management, and future research.

## 2 Pathophysiologic bridges between CTDs and arrhythmias

Arrhythmias in CTDs arise through a set of shared mechanistic bridges that link extracellular matrix (ECM) abnormalities to electrical instability. While individual gene defects vary, most converge on four overlapping pathways (detailed further below): diffuse fibrosis, abnormal valve and annular geometry, altered mechanotransduction, and secondary ion-channel and gap-junction remodeling. These interdependent mechanisms explain why atrial fibrillation (AF), ventricular arrhythmias, and conduction disease appear across CTDs even when left ventricular systolic function is preserved.

### 2.1 Fibrosis as a central substrate

**Figure 1:**
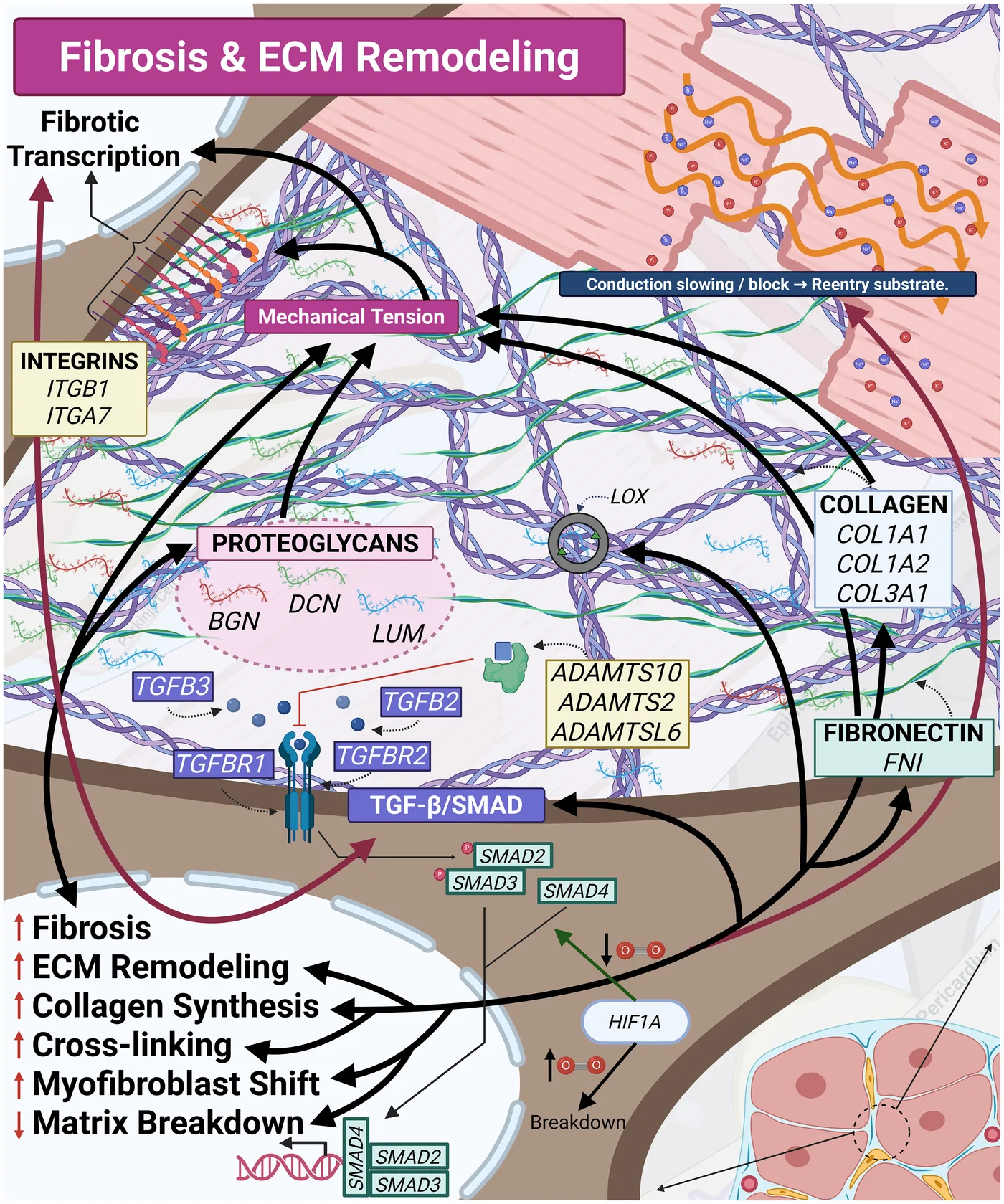
Fibrosis and extracellular matrix remodeling as an arrhythmogenic substrate in CTDs. Activation of canonical and non-canonical TGF-β signaling promotes SMAD2/3-SMAD4 transcriptional programs that upregulate collagens (*COL1A1*, *COL1A2*, *COL3A1*), fibronectin (*FN1*), cross-linking enzymes (*LOX*, *LOXL*), proteoglycans (*BGN*, *DCN*, *LUM*), and matricellular regulators, while also inducing a myofibroblast shift (*ACTA2*) and altering the MMP/TIMP balance. Integrin-mediated mechanotransduction (*ITGB1*, *ITGA7*) and microfibril components (*ADAMTSL6*, *ADAMTS10*, *ADAMTS2*) reinforce a positive feedback loop of ECM stiffening and further TGF-β activation. The result is excessive collagen cross-linking, proteoglycan accumulation, and fibrosis, creating conduction heterogeneity and reentry substrates. The inset illustrates wavefront slowing and fragmentation as fibrotic barriers are encountered, linking molecular remodeling directly to atrial fibrillation, ventricular tachycardia, and sudden cardiac death in CTDs. Gene abbreviations and functional groupings are summarized in Supplementary Table 1.

Excessive TGF-β signaling is a unifying driver of fibrosis in CTDs. Variants in *TGFB2, TGFBR1, TGFBR2,* and *SMAD3* are all associated with fibroblast activation and collagen deposition, creating a substrate for both AF and ventricular tachyarrhythmias (4–6). The profibrotic axis is further reinforced by lysyl oxidase and lysyl oxidase-like enzymes (LOX/LOXL), which stabilize collagen cross-linking and have been associated with atrial fibrosis and AF burden (7). Matricellular proteins such as SPARC also drive collagen assembly and myocardial stiffening, linking pressure overload to arrhythmia risk (8). Hypoxia-responsive pathways integrate with TGF-β through HIF-1*α*, which enhances fibroblast activation and arrhythmogenic remodeling (9). Taken together, these pathways indicate that CTDs may generate arrhythmic substrates not only through structural dilation but also through primary profibrotic signaling. This relationship is summarized schematically in Figure 1, which depicts fibrosis as a substrate for slowed conduction and reentry.

### 2.2 Geometry and mechanical load

**Figure 2:**
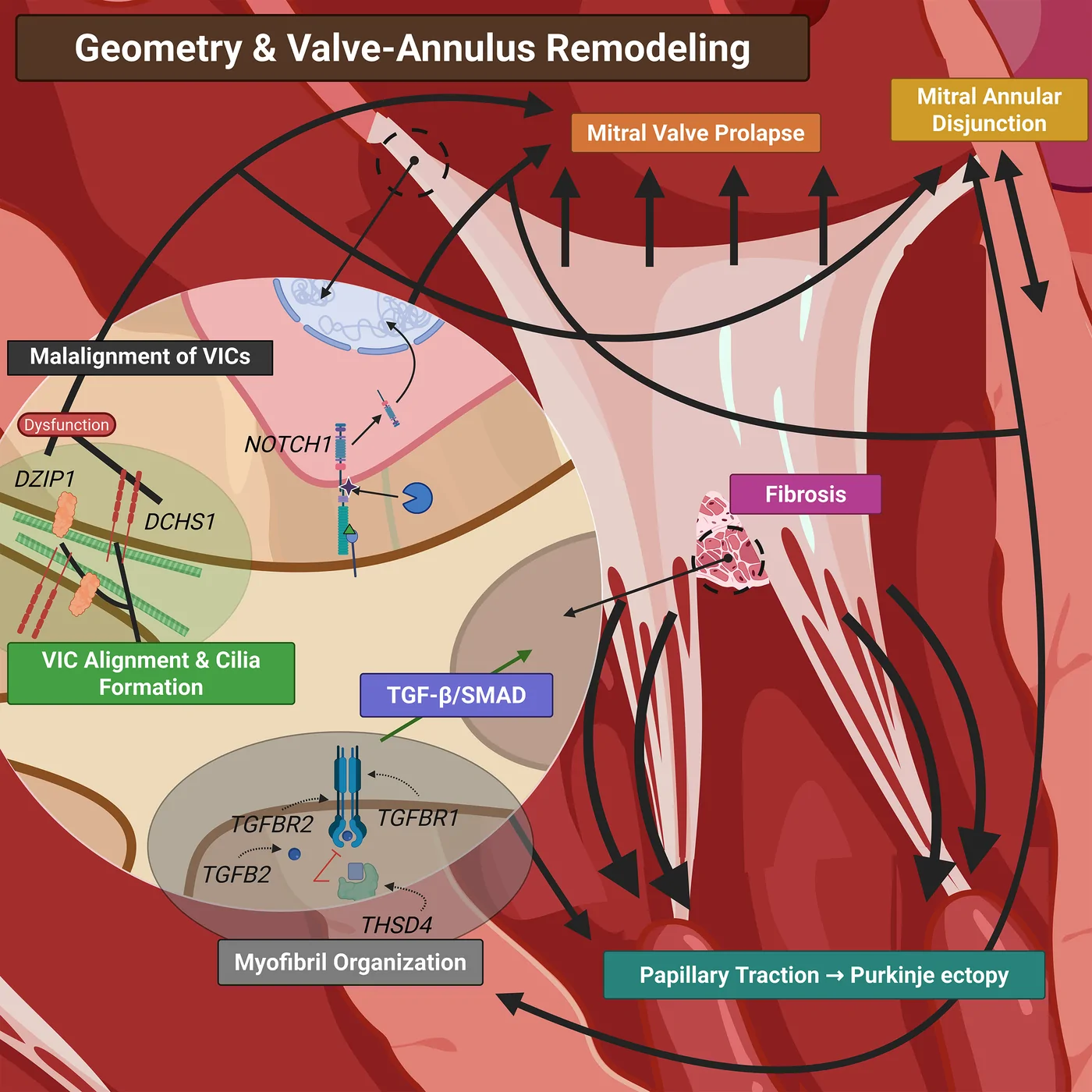
Geometry and valve-annulus remodeling as an arrhythmogenic substrate in connective tissue disorders. Abnormal valve development and annular instability create mechanical substrates for arrhythmias. Developmental misalignment of valve interstitial cells (VICs) through DCHS1 and DZIP1 disrupts cilia formation and planar polarity, impairing mechanosensing. NOTCH1 signaling governs endocardial-to-mesenchymal transition (EMT) and valve morphogenesis; dysregulation leads to leaflet dysmorphogenesis and mitral annular disjunction (MAD). THSD4 stabilizes fibrillin-1 microfibril organization; deficiency weakens the annulus and predisposes to MAD. Together, these pathways promote mitral valve prolapse, annular degeneration, and basal left ventricular fibrosis, which amplify papillary muscle traction and trigger Purkinje ectopy. The result is a geometric and structural substrate for ventricular arrhythmias and sudden cardiac death in CTDs.

Beyond diffuse fibrosis, abnormal valve and annular geometry form another arrhythmogenic bridge. MVP and MAD are associated with increased risk of ventricular arrhythmias and sudden cardiac death, particularly in MFS (1, 10). An important consideration is that the imaging definition of MAD has evolved. Historically, MAD was generally identified as separation of the posterior mitral annulus from the left ventricular myocardium at end systole. More recent dynamic imaging distinguishes true MAD, in which atrial displacement of the posterior leaflet hinge point persists during both diastole and systole, from pseudo-MAD, in which the separation is apparent only during systole. In a contemporary study of 603 patients with myxomatous MVP, true MAD was present in 7%, whereas pseudo-MAD was present in 37%; 84% of patients classified as having MAD using a systolic-only approach would have been reclassified as having pseudo-MAD (11). This distinction may influence reported prevalence and complicate comparisons across studies using different imaging modalities, cardiac phases, measurement thresholds, and anatomical locations. Whether true and pseudo-MAD carry different prognostic implications for ventricular arrhythmias remains incompletely established. *FBN1* variants exemplify this dual mechanism by driving both increased TGF-β signaling and MVP/MAD geometry, with downstream papillary traction and basal left ventricular fibrosis (12). Developmental valve genes reinforce this mechanistic link: *DCHS1* and *DZIP1* cause familial MVP/MAD through planar polarity and ciliary signaling defects (13, 14). *FLNA* and *NOTCH1*, through focal adhesion and endocardial-mesenchymal transition pathways, alter leaflet mechanics and annular stability, amplifying MAD-driven arrhythmic risk (15, 16). Thus, valve geometry in CTDs serves as both a structural and electrophysiologic substrate. This relationship is summarized schematically in Figure 2.

### 2.3 Mechanotransduction: from ECM to nucleus

A fragile or abnormally stiffened ECM in CTDs alters force transmission to cardiomyocytes, destabilizing mechano-electrical homeostasis. Costameric proteins at the sarcolemma transduce strain from the ECM to Z-disc scaffolds and the nuclear lamina, coordinating structural integrity with transcriptional and electrophysiologic responses. Disruption at any point in this chain promotes fibrosis, abnormal ion-channel trafficking, and heightened arrhythmia susceptibility (17, 18). Within this network, several CTD-implicated loci are emerging as modifiers of arrhythmic risk. *LRP1* regulates TGF-β ligand trafficking, integrin-cytoskeleton interactions, and Ca^2+^ handling, linking extracellular signals to both structural and electrical remodeling (19). Integrins α7 and β1 (encoded by *ITGA7* and *ITGB1*) stabilize costameres and modulate ryanodine receptor activity; their dysfunction amplifies fibrotic remodeling and Ca^2+^ instability (20). Together, these pathways illustrate how abnormal ECM stiffness in CTDs propagates inward to nuclear signaling and calcium cycling, generating substrates for atrial and ventricular arrhythmias.

### 2.4 Electrophysiologic consequences of CTD remodeling

**Figure 3:**
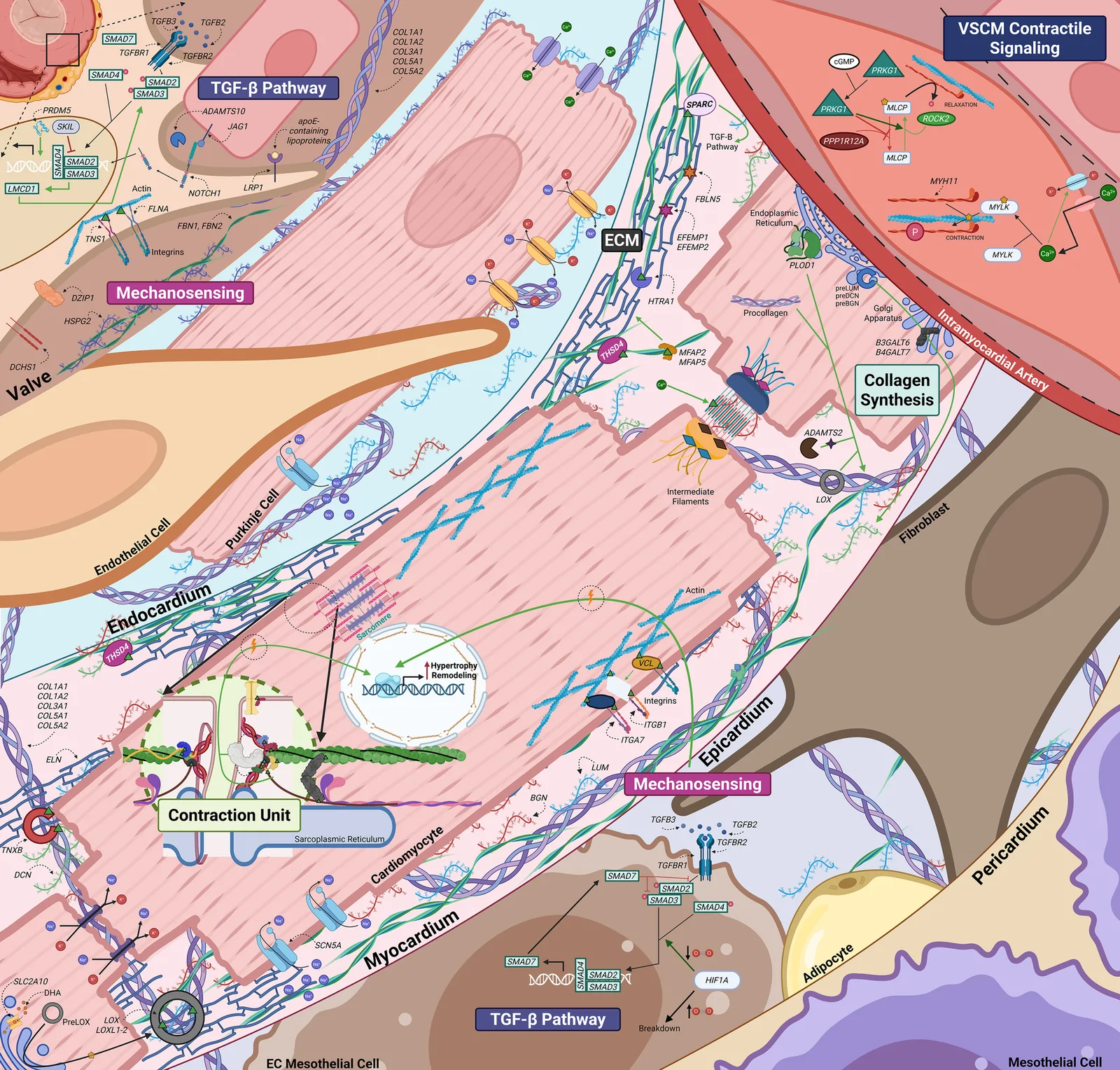
Central illustration of genetic and cellular pathways linking connective tissue disorders to arrhythmogenesis. Schematic representation of the integrated molecular and cellular mechanisms by which connective tissue disorder (CTD) genes influence arrhythmia susceptibility. Pathways converge on extracellular matrix remodeling, valve and annulus structure, cytoskeletal coupling, and ion channel/gap junction function within cardiomyocytes, endothelial cells, fibroblasts, and vascular smooth muscle. Representative genes are mapped to their functional locations, including fibrillar collagens, elastin/fibulins, TGF-β/SMAD signaling, integrins, proteoglycans, desmosomal proteins, ion channels, and contractile cytoskeletal elements. These processes collectively promote fibrosis, altered geometry, impaired mechanotransduction, and repolarization instability that provide substrates for atrial and ventricular arrhythmias. Gene abbreviations and functional groupings are summarized in Supplementary Table 1, and the corresponding symbol and module key is provided in Figure 4.

Arrhythmic risk in CTDs is amplified by downstream remodeling of ion-channel complexes and intercellular junctions. Fibrosis and altered mechanical load likely impair connexin trafficking and gap-junction integrity, slowing conduction and predisposing to reentry (21). Junctional adhesion failure can produce fibrofatty scar and conduction heterogeneity, a process well described in arrhythmogenic cardiomyopathy but paralleled by CTD-driven fibrosis and geometric distortion (22, 23). These secondary electrophysiologic changes suggest that, even in the absence of primary ion-channel or desmosomal mutations, CTDs may reproduce similar downstream vulnerabilities through substrate remodeling, including conduction slowing, repolarization instability, and scar-mediated reentry. This explains why atrial fibrillation, ventricular ectopy, and malignant arrhythmias can emerge in CTDs despite preserved ventricular function. Figure 3 integrates these downstream electrophysiologic consequences with the upstream fibrotic, valve-annular, and mechanotransduction pathways described above.

## 3 Genetic framework of CTD-arrhythmia overlap

**Figure 4:**
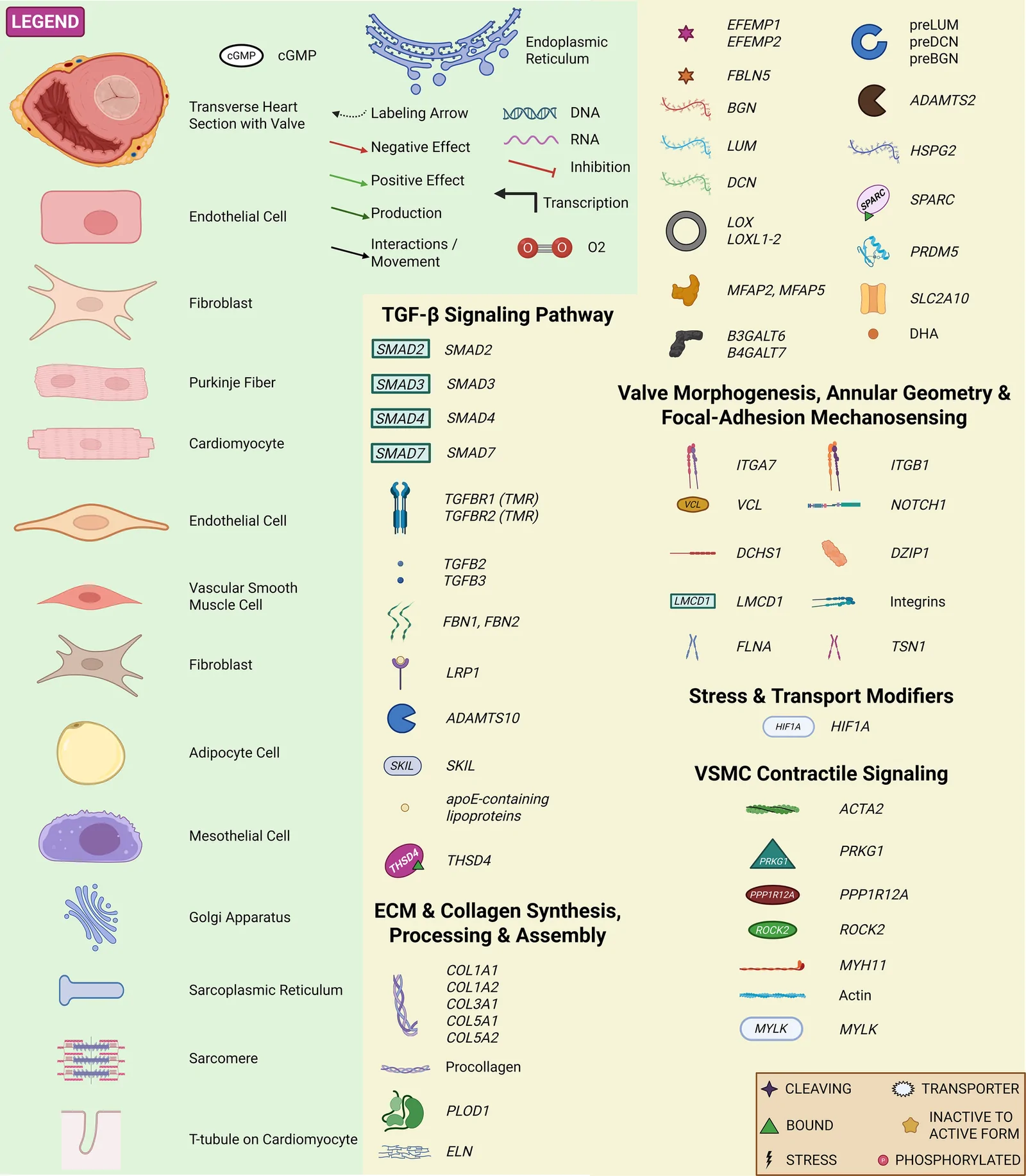
Key to the central illustration. Key to the cell types, organelles, directional arrows, and functional gene groups depicted in Figure 3, including TGF-β signaling; extracellular matrix and collagen pathways; valve morphogenesis and focal-adhesion mechanosensing; vascular smooth muscle cell contractile signaling; cytoskeletal, Z-disc, and nuclear-envelope pathways; cell-cell coupling; ion channels and Ca^2+^ handling; and stress and transport modifiers.

Arrhythmias in connective tissue disorders do not arise from a single mechanism. Rather, they reflect the cumulative effects of abnormal extracellular matrix structure, altered valve and annular mechanics, disturbed mechanotransduction, and downstream electrical remodeling. These processes create the substrate for both atrial and ventricular arrhythmias across a range of syndromes. At the molecular level, each of these pathways is shaped by specific genes involved in matrix integrity, growth factor signaling, valve development, cytoskeletal stability, and cellular coupling. Recent work has broadened this landscape, showing that the genetic basis of connective tissue disease extends well beyond the traditional arteriopathy genes and includes pathways with clear relevance to myocardial and electrophysiologic remodeling (24). In the sections that follow, we focus on CTD-related genes and organize them by biological role to show how diverse molecular defects can converge on shared arrhythmic phenotypes. Figures 1, 2 depict the fibrotic and valve-annular pathways, respectively, whereas Figure 3 provides an integrated central illustration linking the principal genes, cell types, and downstream electrophysiologic effects. Figure 4 provides the corresponding key.

### 3.1 TGF-β and SMAD signaling genes

The TGF-β/SMAD axis is one of the clearest molecular links between connective tissue disorders and arrhythmia risk. Although it is best known for its role in extracellular matrix turnover and vascular remodeling, sustained activation of this pathway also has important myocardial consequences. Within the heart, excess TGF-β signaling promotes fibroblast activation, interstitial collagen deposition, gap-junction disruption, and broader shifts in ion-channel expression. Together, these changes create slow conduction and electrical heterogeneity that favor atrial fibrillation and scar-related ventricular arrhythmias. Clinical observations support this biology: patients with heritable thoracic aortic disease who carry TGF-β pathway variants have been reported to develop both atrial and ventricular arrhythmias, at times even without advanced aortic or valvular disease, suggesting that the myocardium itself may be part of the primary phenotype (6).

Among the ligand genes, *TGFB2* and *TGFB3* encode TGF-β2 and TGF-β3, which activate canonical SMAD signaling through receptor-mediated pathways. Variants affecting these ligands are associated with enhanced profibrotic signaling, connexin remodeling, and conduction heterogeneity, all of which may contribute to atrial fibrillation, ventricular ectopy, and conduction disease (25, 26). *TGFB3* is particularly notable because it underlies Loeys-Dietz syndrome type 5, a condition in which mitral valve prolapse and mitral annular disjunction often intersect with arrhythmic risk. This overlap highlights how TGF-β signaling can influence both myocardial remodeling and valve-annular geometry (25, 27, 28).

A similar pattern is seen at the receptor level. *TGFBR1* and *TGFBR2* encode the type I and type II TGF-β receptors and initiate downstream phosphorylation of SMAD2 and SMAD3 after ligand binding. Pathogenic variants in these receptors shift signaling toward fibrosis, impaired gap-junction integrity, and altered electrophysiologic gene expression. In clinical series, individuals with *TGFBR1* or *TGFBR2* variants, including those with Loeys-Dietz syndrome, have been reported to develop atrial fibrillation, nonsustained ventricular tachycardia, and more clinically significant ventricular arrhythmias, sometimes out of proportion to their overt structural disease (28, 29).

Downstream mediators further shape this phenotype. *SMAD2* and *SMAD4* encode transcriptional effectors of canonical TGF-β signaling and have been linked in experimental models to collagen deposition and conduction slowing (30, 31). Among these genes, *SMAD3* has the strongest clinical association with arrhythmia. Patients with aneurysm-osteoarthritis syndrome, or Loeys-Dietz syndrome type 3, may develop malignant ventricular arrhythmias and sudden cardiac death, supporting the idea that SMAD3-driven fibrosis, junctional remodeling, and ion-channel dysregulation define a particularly high-risk substrate (32, 33). *SMAD7*, by contrast, normally serves as an inhibitory SMAD that restrains TGF-β signaling. Reduced SMAD7 activity may therefore remove an important brake on fibrosis and provide another mechanism by which connective tissue disorders can intensify arrhythmogenic remodeling (31, 34).

Several related genes broaden the relevance of this pathway beyond its core signaling components. *LRP1* encodes low-density lipoprotein receptor-related protein 1, a multifunctional receptor involved in TGF-β trafficking, integrin-cytoskeletal interactions, and calcium handling. *FBN2*, which encodes fibrillin-2, has developmental roles in elastogenesis and regulation of TGF-β availability. Variants in *FBN2* cause congenital contractural arachnodactyly and may be associated with aortic dilation and mitral abnormalities; taken together with experimental evidence of impaired myocardial elastic coupling, these findings suggest a plausible, though still emerging, connection to arrhythmic risk (35, 36).

Additional modifiers may amplify this biology. *HTRA1* encodes a serine protease involved in extracellular matrix turnover and regulation of TGF-β activity, and increased expression has been linked to fibrosis-promoting remodeling (37, 38). *HIF1A*, which encodes hypoxia-inducible factor-1*α*, connects hypoxia and mechanical stress to TGF-β-driven fibroblast activation. Elevated HIF-1*α* has been associated with atrial fibrosis and persistence of atrial fibrillation, suggesting that it may further intensify arrhythmogenic remodeling in susceptible CTD substrates (5, 9).

Overall, disruption across ligands, receptors, intracellular mediators, inhibitory regulators, and pathway modifiers points to a consistent theme: the TGF-β/SMAD network does not simply influence vascular disease in connective tissue disorders but also helps shape a myocardial substrate that is vulnerable to arrhythmia. Among the genes in this pathway, *SMAD3* currently carries the strongest clinical arrhythmic signal, while *TGFB2*, *TGFB3*, *TGFBR1*, *TGFBR2*, *SMAD7*, *FBN2*, *HTRA1*, and *HIF1A* broaden the spectrum of plausible risk. Together, these data support heightened clinical suspicion and consideration of targeted rhythm evaluation in selected patients, even when conventional markers of cardiomyopathy are absent.

### 3.2 Valve and annulus substrate genes

MVP and MAD are among the most recognizable arrhythmogenic phenotypes in connective tissue disorders. Although MVP has traditionally been discussed in relation to valvular dysfunction and progressive regurgitation, it is now clear that the valve-annular complex itself can serve as an arrhythmic substrate. In particular, MVP and MAD have been linked to basal left ventricular fibrosis, Purkinje-related ectopy, and triggered ventricular arrhythmias, even when mitral regurgitation is not severe (1, 2). This has important implications in CTDs, where abnormal valve development, altered annular mechanics, and extracellular matrix fragility may converge to amplify arrhythmic risk.

Several genes help explain why this phenotype is so enriched in CTDs. Among the most direct are *DCHS1* and *DZIP1*, which play key roles in valve development and mechanosensing. *DCHS1* encodes a protocadherin involved in planar cell polarity, and haploinsufficiency has been shown to promote leaflet elongation, thickening, and familial MVP with MAD, all features that align closely with an arrhythmogenic substrate (14, 36, 39). *DZIP1*, a centrosomal and transition-zone protein required for cilia formation, influences valvular extracellular matrix programs through β-catenin signaling. Experimental models and human studies support its role as a causal MVP gene, reinforcing the importance of ciliary and developmental pathways in annular instability and myxomatous degeneration (13, 40). Together, these genes provide some of the clearest mechanistic links between inherited connective tissue abnormalities and the structural phenotype of arrhythmogenic MVP.

Other genes shape this process through effects on valve architecture and mechanical signaling. *FLNA*, which encodes filamin-A, is a well-established mechanosensor and actin-crosslinking protein. Pathogenic variants can cause X-linked myxomatous valvular dystrophy, often with severe multivalvular prolapse and annular abnormalities that may increase arrhythmic vulnerability (16, 41, 42). *TNS1*, encoding tensin-1, has also emerged as an important contributor. As a focal adhesion adaptor linking β-integrins to actin, it helps maintain structural stability at the leaflet-annular interface. Experimental and genetic studies have implicated *TNS1* in MVP, with deficiency promoting annular hypermobility and a fibrosis-prone mechanical substrate (39, 43).

Developmental pathway regulators further broaden this framework. *NOTCH1* is best known for its role in endocardial-to-mesenchymal transition and valve morphogenesis, and while it is not a classic monogenic MVP gene, disruption of this pathway can alter leaflet patterning and annular geometry in ways that plausibly increase arrhythmic risk (15, 44). *LMCD1*, identified through genome-wide and functional studies, appears to act more as a modifier than a primary driver. By influencing transcriptional programs involved in valve development, it may contribute to more subtle forms of leaflet thickening and annular instability that reinforce the MVP/MAD phenotype (43, 45).

What makes these genes clinically important is not simply their association with prolapse, but the way they help explain why some patients develop a distinctly arrhythmogenic form of valve disease. Leaflet redundancy, annular hypermobility, papillary muscle traction, and localized basal fibrosis appear to form a common structural pathway through which these abnormalities translate into ventricular ectopy and malignant arrhythmias. The strongest causal evidence currently supports *DCHS1*, *DZIP1*, and *FLNA*, while *TNS1* and *LMCD1* appear to function more as important modifiers. *NOTCH1*, meanwhile, helps place these findings within a broader developmental context (46).

Taken together, the valve-annulus complex in CTDs should be viewed as more than a bystander structural abnormality. In selected patients, it likely represents a primary arrhythmogenic substrate. This is especially relevant in familial, bileaflet, or syndromic forms of MVP, where regurgitation severity alone may underestimate risk. In these settings, advanced imaging for MAD and fibrosis, along with closer rhythm surveillance, may provide a more meaningful assessment of electrical vulnerability than valve dysfunction alone.

### 3.3 Cytoskeletal and myocyte coupling genes

Cytoskeletal and myocyte coupling pathways form the structural and signaling continuum that translates mechanical load into electrical behavior. This axis includes costameres, Z-disc scaffolds, intermediate filaments, the nuclear lamina, and Ca^2+^-handling units. The recurring failure modes across these systems are (1) conduction system disease from impaired mechanotransduction, (2) scar-dominant ventricular tachyarrhythmias from fibrotic remodeling, and (3) triggered activity from calcium mishandling. Although many insights into these processes derive from arrhythmogenic and dilated cardiomyopathies, these comparisons are used as mechanistic analogies for shared downstream features, including fibrosis, impaired cellular coupling, and conduction heterogeneity. They should not be interpreted as evidence that CTDs and inherited cardiomyopathies share the same causal genetic architecture. CTDs may nevertheless place secondary stress on these pathways through ECM fragility, diffuse fibrosis, and annular hypermobility.

Emerging mechanotransduction genes provide candidate links between CTDs and cytoskeletal remodeling. *LRP1* encodes low-density lipoprotein receptor-related protein-1, which regulates TGF-β trafficking, integrin-cytoskeleton signaling, and Ca^2+^ handling, integrating extracellular strain with intracellular remodeling (47, 48). *ITGA7* and *ITGB1*, encoding α7- and β1-integrins, stabilize costameres and modulate ryanodine receptor activity. Dysregulation of these integrins amplifies fibrotic remodeling and Ca^2+^ instability, providing a plausible substrate for arrhythmogenicity in CTDs (49). *VCL*, encoding vinculin, links integrins to actin at costameres; loss of vinculin-mediated stabilization in experimental models produces conduction abnormalities and arrhythmic death, underscoring its role as an emerging CTD-relevant mechanosensor (50).

Beyond focal adhesion pathways, vascular contractility genes highlight a direct CTD connection to arrhythmia risk. *MYH11*, encoding smooth muscle myosin heavy chain, is a canonical aortopathy gene in which variants cause familial thoracic aortic aneurysm/dissection and patent ductus arteriosus. While arrhythmias are not a primary phenotype, impaired arterial mechanics increase LV afterload and myocardial fibrosis, indirectly predisposing to atrial and ventricular arrhythmias (51, 52). *MYLK*, encoding myosin light-chain kinase, is another established HTAD gene associated with thoracic aortic dissection, in which altered vascular wall stress may similarly raise arrhythmogenic susceptibility (53, 54). *PRKG1*, encoding protein kinase G type I, is a gain-of-function HTAD gene; *PKG1* expression regulates hypertrophy, fibrosis, and calcium handling, suggesting a mechanistic plausibility for arrhythmogenic remodeling in CTD overlap (55, 56).

Taken together, these data suggest that chronic load imbalance and ECM fragility in CTDs can disturb cytoskeletal and myocyte-coupling pathways even when the initiating variant is not a primary cardiomyopathy gene. Genes such as *LRP1*, *ITGA7*, *ITGB1*, and *VCL* represent emerging mechanotransduction amplifiers, while vascular contractility loci (*MYH11*, *MYLK*, *PRKG1*) provide direct genetic anchors. Collectively, these mechanisms broaden the spectrum of CTD-associated arrhythmic risk beyond valve or ECM substrates to include secondary stress on cytoskeletal and vascular contractility pathways.

### 3.4 Extracellular matrix and structural matrix genes

ECM genes are central to the mechanical and electrical phenotype of CTDs, determining tissue tensile strength, elasticity, and mechanosensing. Disruption of these loci weakens structural integrity, amplifies fibrosis, and alters annular geometry, producing substrates for atrial fibrillation, conduction heterogeneity, and ventricular arrhythmias.

Collagen genes exemplify this vulnerability. *COL3A1*, mutated in vascular EDS, and *COL5A1/2*, causal in classical EDS, disrupt fibrillogenesis, leading to tissue fragility, myxomatous valve change, and diffuse fibrosis that lower arrhythmic thresholds (57, 58). *PLOD1* deficiency in kyphoscoliotic EDS impairs collagen cross-linking, promoting patchy fibrosis and anisotropic conduction (59). Cross-linking enzymes, including the *LOX*/*LOXL* family, reinforce this axis. Their upregulation has been linked to myocardial fibrosis, conduction slowing, and reentry in broader cardiovascular models, although direct CTD-specific arrhythmia evidence and therapeutic implications remain limited (7, 60, 61).

Elastic fiber defects also contribute. *ELN* haploinsufficiency, as seen in Williams-Beuren syndrome, stiffens arteries and predisposes to atrial fibrillation (62, 63). *EFEMP2* (fibulin-4) and *FBLN5* (fibulin-5) variants cause cutis laxa with vascular tortuosity and aneurysms, while experimental models show impaired elastogenesis induces fibrosis and connexin disarray, linking directly to arrhythmogenic remodeling (64–66). Emerging evidence suggests *EFEMP1* (fibulin-3) similarly weakens elastic networks; reduced expression in aneurysm tissue and GWAS associations with vascular traits highlight its potential role as an arrhythmia-modifying locus (67, 68).

Fibrillin and microfibril-associated proteins form another axis. *FBN1* deficiency in Marfan syndrome drives excess TGF-β activation and MVP/MAD geometry, both strongly associated with malignant ventricular arrhythmias (12, 69). Related microfibrillar proteins (*MFAP2*, *MFAP5*) regulate growth-factor signaling and elastin assembly, with variants linked to familial TAAD and arrhythmogenic remodeling (52, 70, 71). *THSD4* (ADAMTSL6) loss destabilizes fibrillin architecture and encourages MVP-like mechanics, while *ADAMTS10* defects in Weill-Marchesani syndrome perturb microfibril stiffness, indirectly predisposing to arrhythmia (72–74).

Proteoglycans and linkeropathies add further layers. *BGN* (encodes biglycan) and *HSPG2* (encodes perlecan) regulate fibrillogenesis, fibroblast activation, and conduction maturation; their dysregulation promotes fibrosis and heterogeneity (4, 75, 76). *TNXB* deficiency in classical-like EDS destabilizes annular structure and myxomatous remodeling (77). Linkeropathies due to *B3GALT6* and *B4GALT7* impair proteoglycan biosynthesis, reducing ECM hydration and anisotropy thresholds (78–80). Small leucine-rich proteoglycans such as *DCN* (decorin) and *LUM* (lumican) are emerging contributors: decorin restrains TGF-β signaling and fibrosis, while lumican regulates fibril spacing and stiffness; deficiency of either accelerates fibrotic remodeling and atrial arrhythmias (81–83).

Other ECM regulators emphasize transcriptional and matricellular control of fibrosis. *ADAMTS2* variants in dermatosparaxis EDS disrupt collagen fibril maturation, favoring micro-scarring and arrhythmia (84). *SLC2A10*, causal in arterial tortuosity syndrome, alters oxidative stress and TGF-β/ECM signaling, indirectly shifting loading and fibrosis thresholds (85). *PRDM5* encodes a chromatin regulator of ECM gene expression, and *SPARC* encodes a matricellular protein essential for collagen deposition, both amplify fibrotic cascades when dysregulated, increasing conduction heterogeneity and arrhythmogenic remodeling (34, 37, 38, 86).

Taken together, ECM and structural matrix genes illustrate how CTDs destabilize microfibrils, elastic fibers, proteoglycans, and collagen networks. Core loci such as *FBN1*, *COL3A1*, and *COL5A1/2* define the underlying connective-tissue phenotype, while modifiers including *LOX*, *ELN*, *EFEMP1*, *BGN*, *DCN*, *LUM*, *HSPG2*, *TNXB*, *B3GALT6*/7, *PRDM5*, and *SPARC* may broaden the arrhythmic substrate through fibrosis, annular instability, and conduction heterogeneity. Across these diverse pathways, the shared output is diffuse fibrosis, annular laxity, and reentrant vulnerability, explaining why CTDs predispose to arrhythmias even in the absence of overt cardiomyopathy.

### 3.5 Ion channels, gap junctions, and electrophysiologic modifiers

Although the CTDs emphasized in this review are generally not primary ion-channel or desmosomal disorders, their arrhythmogenic substrates may converge on the same downstream machinery. Fibrosis, mitral annular disjunction, and altered mechanotransduction remodel sodium, calcium, and potassium channel complexes as well as gap junctions and intercalated disc structures. These changes slow conduction, reduce repolarization reserve, and destabilize calcium cycling, creating the substrates for atrial fibrillation, triggered ventricular ectopy, and malignant reentrant arrhythmias (87–98). Gap-junction dispersion and desmosomal remodeling amplify this process, fostering conduction block and scar anisotropy similar to arrhythmogenic cardiomyopathy (21, 23).

Pacemaker currents are also vulnerable. In CTDs, diffuse fibrosis and altered autonomic signaling can impair sinoatrial node function, producing bradyarrhythmia and atrial arrhythmia phenocopies of primary pacemaker channel disease (99, 100). Collectively, these processes illustrate how CTD-related structural remodeling secondarily stresses electrophysiologic nodes, phenocopying channelopathy and desmosomal syndromes and explaining why arrhythmias often arise despite preserved ventricular function.

## 4 Clinical phenotypes by Major CTDs

Recent comparative data suggest that arrhythmic phenotypes differ across the major heritable aortopathies. In a multicenter cohort of 1,032 adults with Marfan syndrome, Loeys-Dietz syndrome, or vascular Ehlers-Danlos syndrome, atrial fibrillation or flutter was documented in 23.8%, 30.9%, and 11.4%, respectively (101). A separate propensity-matched international database analysis reported ventricular tachycardia in 2.6% of patients with Marfan syndrome compared with 0.9% of those with vascular Ehlers-Danlos syndrome (102). These estimates were derived from selected clinical and administrative-database populations rather than population-based surveillance, but they provide useful comparative context for the syndrome-specific evidence summarized below and in Table 1.

**Table 1:** Clinical studies reporting arrhythmic phenotypes and related structural findings in connective tissue disorders.

Disorder	Study population and design	Cohort size	Reported arrhythmic findings	Structural or imaging findings	Principal limitations
Genetically confirmed HTAD	International multicenter retrospective cohort of patients with pathogenic or likely pathogenic variants in *FBN1*, TGF-β signaling genes, or *ACTA2*	3,219 screened; 101 with non-aortic cardiac disease	Non-aortic cardiac disease was identified in 3.1% of screened patients. Among 142 reported manifestations, 47 (33%) were AF or AFL and 27 (19%) were VT, VF, or SCD.	Prior cardiovascular surgery was present in 80% and severe valvular disease in 58%; 18% developed non-aortic cardiac disease without either.	Rhythm surveillance was not standardized. The percentages for AF/AFL and VT/VF/SCD describe the distribution of reported manifestations rather than prevalence among all patients with HTAD.
MFS, LDS, and vEDS	Retrospective comparative cohort; formally published conference abstract	1,032	AF or AFL was documented in 23.8% of patients with MFS, 30.9% with LDS, and 11.4% with vEDS.	Not reported in the published abstract.	Abstract-level reporting with limited methodological detail; selected clinical populations without a general-population comparator.
MFS	Nationwide retrospective analysis of hospitalizations in the National Inpatient Sample from 2005 through 2014	12,079 hospitalizations	An arrhythmia was coded in 1,893 hospitalizations (15.7%). A co-diagnosis of POTS was present in 4.9%. VT was associated with the highest in-hospital mortality among the rhythm categories examined.	Structural and imaging findings were not available.	Hospitalization-level administrative data based on ICD-9-CM coding; repeat hospitalizations could not be distinguished from unique patients, and the findings should not be interpreted as population-level prevalence.
MFS	Retrospective cohort of genetically confirmed MFS with echocardiography and ambulatory rhythm assessment	142	Ventricular ectopy and nonsustained VT were more frequent in patients with MAD. Sustained VT or SCD occurred exclusively among patients with MAD.	MAD was present in approximately one-third of patients and was closely associated with MVP. More extensive MAD was also associated with mitral valve intervention and aortic events.	Single referral cohort with few major arrhythmic events; MAD was assessed using the imaging definition applied at the time of the study.
MFS	Cross-sectional, single-center CMR study of patients with a pathogenic or likely pathogenic *FBN1* variant	32	Twenty-four-hour Holter data were available in 28 patients, but the study was not designed to establish arrhythmia prevalence or prospective arrhythmic risk.	Diffuse myocardial fibrosis was identified in 6 patients (21%); focal fibrosis was absent. MAD was associated with larger ventricular volumes and lower ventricular function.	Small, cross-sectional imaging cohort without a control group or longitudinal arrhythmic outcomes.
MFS	Multicenter retrospective study of patients undergoing catheter ablation for AF or AFL	67 underwent ablation; 41 underwent rhythm-targeted AF ablation	Among patients undergoing AF ablation, 34 of 41 (82.9%) experienced recurrent atrial arrhythmia and 20 (48.8%) underwent multiple procedures. Complications occurred in 19 of 67 patients (28.4%) undergoing ablation.	Echocardiographic characteristics were collected, but imaging predictors were not the primary focus.	Highly selected procedural cohort without a non-MFS control group; ablation indications and procedural approaches were heterogeneous.
MFS vs. vEDS	Propensity-matched international administrative-database analysis; formally published conference abstract	1,091 patients per matched group	VT was documented in 2.6% of patients with MFS and 0.9% of patients with vEDS.	Structural and imaging findings were not available.	Reliance on diagnostic coding without manual rhythm adjudication; residual confounding and limited methodological detail in the published abstract.
LDS	Multicenter retrospective cohort evaluating atrial, ventricular, and conduction abnormalities	94	Atrial arrhythmias occurred in 31.9%, including AF in 28.7%, AFL in 12.7%, and SVT in 19.1%. Ventricular arrhythmias occurred in 20.2%, and conduction abnormalities occurred in 37.2%.	MVP and MAD were present in 35.1% and 11.7%, respectively, and were each associated with ventricular arrhythmias.	Referral cohort with a high burden of cardiovascular surgery; rhythm ascertainment depended on available clinical testing and documentation.
LDS	Multicenter retrospective analysis of postoperative outcomes and valvular involvement	94	New-onset postoperative arrhythmias occurred in more than 40% of patients, predominantly AF or AFL.	Mitral and tricuspid valve disease were common. Mechanical valve recipients experienced substantial anticoagulation-associated bleeding.	Surgically enriched population; postoperative findings should not be interpreted as arrhythmia prevalence in unselected patients with LDS.
LDS	Retrospective CMR cohort of adults undergoing routine aortic imaging	46	Sustained VT occurred only in the MAD group, although the number of severe arrhythmic events was small.	MAD was present in 17 patients (37%) and was associated with younger age at aortic events and progressive aortic root enlargement.	Small, CMR-selected cohort with few clinical events and limited power to define arrhythmic risk.
EDS and HSD	Retrospective study of symptomatic patients evaluated with an implantable loop recorder	116 evaluated; 31 received an implantable loop recorder	Among monitored patients, symptomatic sinus tachycardia occurred in 51.6%, PVCs in 25.8%, SVT in 19.4%, and VT in 3.2%. Findings prompted PVC ablation in one patient and ICD implantation in one patient with symptomatic VT.	Structural imaging findings were not a primary study outcome.	Symptom-selected population composed predominantly of women with hypermobile EDS or HSD; findings are not generalizable to all EDS subtypes or asymptomatic patients.
Williams syndrome	Retrospective pediatric cohort undergoing ambulatory ECG monitoring	74	Arrhythmias were identified in 9 patients (12%). Atrial tachycardia was the most common arrhythmia, occurring in 6 patients (8% of the total cohort).	Arrhythmias were not associated with the severity of structural heart disease.	Pediatric tertiary-care cohort selected for ambulatory monitoring; small number of arrhythmic events.
Osteogenesis imperfecta	Systematic review of case-control studies and cohort series	22 studies	The only population-based study addressing atrial arrhythmias reported a higher risk of AF or AFL in osteogenesis imperfecta than in matched controls, with a subhazard ratio of 1.7.	Valvular disease and larger aortic root dimensions were reported more frequently in patients with osteogenesis imperfecta than in controls.	AF and AFL were evaluated in only one included study. Included studies were heterogeneous in age, osteogenesis imperfecta subtype, cardiovascular assessment, and design.

Data are presented as reported by the individual studies. Because the included populations differed in age, referral setting, surgical history, monitoring intensity, imaging methods, and outcome definitions, estimates should not be compared directly or interpreted as population-level prevalence. Percentages reported among patients with identified non-aortic cardiac disease, patients undergoing ablation, or symptomatic patients receiving an implantable loop recorder do not represent prevalence within the broader disorder. Hospitalization-based estimates may include repeated admissions by the same patient. The comparative MFS, LDS, and vEDS cohort and the MFS-vs.-vEDS ventricular tachycardia analysis were formally published conference abstracts. POTS was a co-primary outcome in the nationwide MFS study but is an autonomic disorder rather than a primary cardiac arrhythmia.

Sources: Genetically confirmed HTAD cohort, reference 25; comparative MFS, LDS, and vEDS cohort, reference 101; nationwide MFS inpatient study, reference 106; MFS MAD cohort, reference 69; MFS CMR fibrosis cohort, reference 107; MFS ablation cohort, reference 120; MFS-vs.-vEDS ventricular tachycardia analysis, reference 102; comprehensive LDS arrhythmia cohort, reference 110; LDS postoperative cohort, reference 104; LDS CMR cohort, reference 111; EDS and HSD loop-recorder cohort, reference 114; Williams syndrome cohort, reference 115; and osteogenesis imperfecta systematic review, reference 116.

AF, atrial fibrillation; AFL, atrial flutter; CMR, cardiac magnetic resonance; ECG, electrocardiography; EDS, Ehlers-Danlos syndrome; HSD, hypermobility spectrum disorder; HTAD, heritable thoracic aortic disease; ICD, implantable cardioverter-defibrillator; LDS, Loeys-Dietz syndrome; MAD, mitral annular disjunction; MFS, Marfan syndrome; MVP, mitral valve prolapse; POTS, postural orthostatic tachycardia syndrome; PVC, premature ventricular contraction; SCD, sudden cardiac death; SVT, supraventricular tachycardia; vEDS, vascular Ehlers-Danlos syndrome; VF, ventricular fibrillation; VT, ventricular tachycardia.

Interpretation of these clinical frequencies must also account for the substantial cumulative surgical burden in MFS and LDS. In a multicenter cohort of 783 adults with MFS, 58.0% underwent at least one cardiac operation, and 41.0% of operated patients required redo sternotomy (103). Similarly, 64.9% of patients in a multicenter LDS cohort underwent cardiovascular surgery, with frequent postoperative complications and new-onset arrhythmias (104). Although these observational studies cannot determine the extent to which surgery causes subsequent rhythm disease, they suggest that cumulative operative exposure and residual or progressive valvular disease may modify the observed arrhythmic phenotype. This contribution should be considered alongside, rather than instead of, the intrinsic myocardial and valve-annular substrates described in patients without prior surgery.

A broader aortopathy comparator supports this multifactorial interpretation. In a multicenter cohort of 3,989 adults with chart-confirmed nonsyndromic thoracic aortic disease after exclusion of syndromic CTDs, atrial fibrillation, atrial flutter, supraventricular tachycardia, ventricular tachycardia, and ventricular fibrillation were documented in 21.3%, 7.0%, 15.1%, 5.2%, and 1.0% of patients, respectively (105). Men had higher frequencies of atrial fibrillation (24.0% vs. 14.6%) and atrial flutter (8.3% vs. 3.7%), whereas women had more supraventricular tachycardia (19.5% vs. 13.4%); ventricular tachycardia and ventricular fibrillation did not differ significantly by sex (105). Because rhythm diagnoses were recorded as ever documented and their timing relative to aortic surgery could not be established, these data cannot determine whether arrhythmias reflect the aortic disease substrate, associated cardiovascular comorbidity, or procedural exposure. Nevertheless, they demonstrate that clinically relevant rhythm disease is also present outside syndromic CTDs and provide useful context for interpreting the MFS, LDS, and vEDS findings.

### 4.1 Marfan syndrome

Arrhythmias are becoming increasingly recognized as an important non-aortic manifestation of Marfan syndrome (MFS), complementing the well-known burden of aortic dilation and dissection. Across large heritable thoracic aortic disease (HTAD) cohorts, arrhythmias and impaired myocardial function have been reported primarily in patients with *FBN1* pathogenic variants, suggesting that MFS carries a distinct arrhythmogenic phenotype beyond surgical or valvular sequelae (25). In this international study, atrial fibrillation/flutter accounted for one-third of arrhythmic presentations, while ventricular tachyarrhythmias and sudden cardiac death comprised nearly one-fifth, with a notable subset occurring in the absence of prior surgery or severe valve disease. These findings underscore that electrical instability in MFS is not solely secondary to advanced structural disease.

Complementing these referral-cohort observations, broader administrative data provide additional epidemiologic context. In a nationwide analysis of 12,079 MFS hospitalizations from 2005 through 2014, an arrhythmia was coded in 1,893 hospitalizations (15.7%), and ventricular tachycardia was associated with the highest in-hospital mortality among the rhythm categories examined (106). Because the unit of analysis was hospitalization rather than an individual patient and ascertainment relied on administrative coding, these findings are not directly comparable with the multicenter cohort estimates above and should not be interpreted as population-level prevalence.

Cardiac magnetic resonance (CMR) studies have provided additional mechanistic insight. In a dedicated MFS cohort with confirmed *FBN1* variants, diffuse extracellular volume expansion consistent with fibrosis was observed in approximately 20% of patients, although focal fibrosis was absent (107). While fibrosis burden was not directly correlated with overt systolic dysfunction, patients with mitral annular disjunction (MAD) demonstrated larger ventricular volumes and reduced ejection fraction, suggesting that geometry and fibrotic remodeling interact to shape arrhythmic risk. The clinical importance of MAD in MFS has been highlighted in multiple series. In a cohort of 142 genetically confirmed patients, MAD was present in one-third when assessed using the end-systolic definition applied in that study and was closely associated with mitral valve prolapse, ventricular ectopy, and nonsustained VT (69). Importantly, arrhythmic events and the need for mitral valve surgery occurred exclusively in patients with MAD, and those with more extensive MAD (>10 mm) also exhibited higher rates of aortic events. These findings reinforce MAD as a marker of adverse outcomes in MFS, extending beyond valve degeneration to arrhythmogenic remodeling. Case reports and small pediatric cohorts corroborate this relationship, including ventricular arrhythmias in children with MFS and MAD (12, 108).

At the mechanistic level, myxomatous degeneration of the mitral valve represents the structural substrate linking *FBN1* deficiency to MAD and ventricular arrhythmias. While mitral valve prolapse (MVP) affects approximately 2% to 3% of the general population, it is enriched in syndromic forms such as MFS, where genetic perturbations amplify leaflet thickening and annular instability (39). The resultant papillary traction and basal left ventricular fibrosis create a substrate for malignant arrhythmias, independent of regurgitation severity. Taken together, these data suggest that MFS is not solely a vascular disorder but may also include an arrhythmogenic myocardial and valve-annular phenotype. MAD is among the most consistently reported imaging markers of arrhythmic vulnerability in MFS, with fibrosis serving as a potential substrate amplifier. Clinically, these findings support considering targeted rhythm surveillance, CMR assessment of fibrosis, and MAD characterization in selected patients, particularly those with symptoms, ventricular ectopy, or complex valve phenotypes.

### 4.2 Loeys-Dietz syndrome

Loeys-Dietz syndrome (LDS) is a CTD caused by pathogenic variants in the TGF-β signaling pathway, characterized by aggressive aortic disease, systemic features, and multisystem involvement. While vascular complications remain the primary determinant of morbidity and mortality, emerging data underscore that arrhythmias and myocardial dysfunction also contribute significantly to the LDS phenotype. In addition to atrial and ventricular septal defects, patent ductus arteriosus, and valve pathology, atrial fibrillation and cardiomyopathy have been reported as part of the broader cardiovascular spectrum (109).

Dedicated rhythm phenotyping has demonstrated that arrhythmias in LDS extend beyond the postoperative setting. In a multicenter cohort of 94 patients, atrial arrhythmias were present in 31.9%, ventricular arrhythmias in 20.2%, and conduction abnormalities in 37.2%; atrial fibrillation was the predominant atrial phenotype, and many rhythm abnormalities occurred independently of recent cardiac surgery (110). Mitral valve prolapse and mitral annular disjunction were each strongly associated with ventricular arrhythmias, linking the broader clinical phenotype to the valve-annular substrate described above (110).

Postoperative remodeling nevertheless remains clinically important. In a complementary multicenter analysis focused on surgical outcomes, new-onset arrhythmias occurred in more than 40% of patients, primarily atrial fibrillation or flutter, alongside a high burden of mitral and tricuspid valve disease (104). Mechanical valve recipients also experienced substantial bleeding risk during anticoagulation, underscoring the competing hazards that complicate rhythm management following cardiovascular surgery (104).

Complementing these broader clinical data, cardiac magnetic resonance studies have identified MAD as a potential imaging and prognostic marker in LDS. In a dedicated cardiac MRI cohort using the systolic imaging definition applied in that study, MAD was present in approximately one-third of patients, associated with progressive aortic root dilation, younger age at first aortic event, and a correlation with the need for valve-sparing root replacement (111). Importantly, sustained ventricular tachycardia occurred exclusively in the MAD group, linking annular instability to malignant electrical outcomes. These findings mirror observations in Marfan syndrome and support MAD quantification as a clinically relevant imaging biomarker in LDS.

Taken together, LDS demonstrates a broad arrhythmic phenotype encompassing atrial arrhythmias, ventricular arrhythmias, and conduction disease, much of which is not confined to the postoperative setting. Valve pathology, MAD, and surgical remodeling appear to act as important modifiers rather than complete explanations for this burden. These findings support surveillance strategies that integrate rhythm assessment, advanced imaging for valve-annular abnormalities, and careful perioperative follow-up while accounting for the bleeding and procedural risks inherent to the connective tissue substrate.

### 4.3 Ehlers-Danlos syndromes (EDS)

The Ehlers-Danlos syndromes (EDS) encompass a heterogeneous group of connective tissue disorders, with vascular EDS (vEDS, *COL3A1* variants) representing the most severe phenotype because of arterial rupture and organ fragility. In contrast, classical and hypermobile forms (cEDS and hEDS) are more prevalent but remain less well characterized from a cardiovascular perspective. Evidence regarding arrhythmias remains limited and heterogeneous, consisting primarily of case reports, small observational cohorts, symptom-selected monitoring studies, and recently published comparative analyses. Collectively, these data suggest that myocardial fragility, conduction abnormalities, and procedural complications may represent underrecognized contributors to morbidity, while also emphasizing that the rhythm phenotype differs considerably among EDS subtypes.

Beyond the recent comparative analyses, individual reports in vEDS describe atrial fibrillation and conduction abnormalities even in the absence of advanced structural disease (57). More severe arrhythmias have also been documented: a case of vEDS demonstrated malignant complications after routine ICD placement, underscoring how myocardial and vascular fragility complicate rhythm device therapy (112). These examples highlight the dual challenge in vEDS of both arrhythmia risk and the dangers inherent in standard interventional management.

In hypermobile and classic EDS, symptomatic ventricular ectopy has been described, with limited success using conventional antiarrhythmics. One case highlighted dramatic suppression of frequent PVCs with flecainide after failure of multiple agents, illustrating both the therapeutic challenges and potential role of tailored pharmacologic strategies when invasive procedures are deemed unsafe due to vascular risk (113). Broader observational data reinforce the arrhythmic burden in hEDS: a recent study employing implantable loop recorders in EDS and hypermobility spectrum disorder revealed frequent symptomatic arrhythmias including sinus tachycardia, supraventricular tachycardia, PVCs, and, in rare cases, ventricular tachycardia. Importantly, device monitoring clarified the distinction between postural orthostatic tachycardia syndrome (POTS) and true arrhythmias, guided therapy efficacy, and in select cases prompted escalation to ablation or ICD implantation (114).

Taken together, while systematic studies are lacking, the available literature suggests that EDS patients across subtypes may be predisposed to atrial arrhythmias, frequent ventricular ectopy, and, occasionally, malignant ventricular arrhythmias. The combination of myocardial fragility and vascular risk complicates the application of standard interventional strategies such as ablation or device therapy. This highlights the importance of noninvasive rhythm surveillance, cautious selection of antiarrhythmic pharmacotherapy, and individualized monitoring approaches. Future studies are needed to clarify the true arrhythmic prevalence in EDS subtypes and to develop safe, tailored management strategies for this fragile population.

### 4.4 Rare connective tissue disorders (CTDs)

Beyond MFS, LDS, and EDS, several rarer connective tissue disorders also appear to carry cardiovascular and potentially arrhythmic manifestations, although the available literature remains limited. In most cases, the evidence comes from case reports, small series, or observational data rather than dedicated rhythm studies. Even so, the patterns that have emerged suggest that myocardial fragility, abnormal valve structure, and broader connective tissue instability may contribute to electrical vulnerability in at least a subset of these disorders.

Among the rarer CTDs, Williams syndrome currently has some of the clearest clinical data linking the disorder to arrhythmia. In a retrospective cohort of patients undergoing ambulatory monitoring, arrhythmias were identified in 12%, most commonly atrial tachycardia, alongside repolarization abnormalities and a substantially increased risk of sudden cardiac death (115). These findings suggest that arrhythmias in Williams syndrome are not merely incidental but may represent a clinically meaningful component of the cardiovascular phenotype. Osteogenesis imperfecta (OI) also has more substantive evidence than many other rare CTDs. A recent systematic review demonstrated increased prevalence of atrial fibrillation, valvular disease, and larger aortic root dimensions compared with controls, supporting the concept that abnormal collagen type I may affect both structural and electrical aspects of cardiac disease (116).

For other rare CTDs, the signal is more limited but still noteworthy. Cutis laxa syndromes are primarily recognized for skin laxity and vascular fragility, yet congenital forms can also involve severe cardiopulmonary disease. Published case reports describe multivalvular involvement requiring surgical management, raising the possibility that altered valve structure and connective tissue weakness may also create a substrate for conduction abnormalities, even though dedicated arrhythmia series are lacking (117). Weill-Marchesani syndrome (WMS), classically viewed as an ocular and skeletal disorder, has also been associated with prolonged QTc intervals (118). Overt arrhythmias have not been well characterized, but the presence of repolarization abnormalities suggests that electrical involvement may be underrecognized rather than absent.

Arterial tortuosity syndrome (ATS), caused by *SLC2A10* variants, is another example of a disorder in which vascular pathology dominates the clinical picture, yet occasional electrical manifestations have been reported. A case report describing atrial fibrillation in a patient with ATS suggests that even predominantly arterial connective tissue disorders may have underappreciated arrhythmic potential (119). Although such reports remain isolated, they are important because they broaden the discussion beyond the better-described syndromes and point toward shared mechanisms that may extend across the CTD spectrum.

Overall, rare CTDs remain markedly understudied with respect to arrhythmias. Williams syndrome and osteogenesis imperfecta provide the strongest current evidence that rhythm disturbances may be part of the broader phenotype, whereas cutis laxa, Weill-Marchesani syndrome, and arterial tortuosity syndrome suggest possible but still poorly defined risk. What ties these disorders together is the likelihood that matrix instability, vascular fragility, and abnormal valvular or myocardial structure can create a substrate for electrical disease. More systematic surveillance and dedicated mechanistic studies will be needed to define the true prevalence of arrhythmias in these conditions and determine whether targeted monitoring can reduce adverse outcomes.

## 5 Clinical translation: diagnostics, management, and future directions

As interest in arrhythmias in connective tissue disorders continues to grow, the challenge is no longer simply recognizing that these patients may be at risk but determining how that risk should influence clinical care. Advanced imaging, rhythm monitoring, and genetic context may help refine suspicion in selected patients, but none is supported strongly enough to justify a uniform surveillance strategy across all CTDs. The clinical implications outlined below should therefore be interpreted in the context of the limitations of the available evidence discussed in Section 5.6.

### 5.1 Advanced imaging

Advanced cardiac imaging may help identify patients with CTDs who carry a more arrhythmogenic phenotype. In Marfan syndrome, cardiac magnetic resonance imaging has demonstrated diffuse extracellular volume expansion in a subset of patients, consistent with myocardial fibrosis, although focal scar was not seen and clear correlations with systolic dysfunction were limited (107). In Loeys-Dietz syndrome, cardiac MRI has also identified mitral annular disjunction in more than one-third of patients, with associations with earlier aortic events and isolated cases of sustained ventricular tachycardia (111). Accordingly, MAD assessment should account for the full cardiac cycle, with careful tracking of the posterior leaflet hinge point from diastole through systole to avoid misclassifying systolic-only pseudo-MAD as persistent annular disjunction (11). Taken together, these observations suggest that diffuse fibrosis and MAD may serve as markers of increased electrical vulnerability in selected patients. At the same time, the evidence is not yet strong enough to support routine screening solely for arrhythmia risk. For now, advanced imaging is probably most useful when symptoms, ectopy, valve abnormalities, or a concerning phenotype raise suspicion for a more complex substrate.

### 5.2 Ambulatory rhythm monitoring

Ambulatory rhythm monitoring is another important tool, particularly because arrhythmias in CTDs may be intermittent, underrecognized, or initially attributed to nonarrhythmic symptoms. In Marfan syndrome, patients with MAD have shown higher rates of ventricular ectopy and nonsustained ventricular tachycardia on Holter monitoring (69). In Williams syndrome, ambulatory ECG monitoring identified primary arrhythmias in approximately 12% of patients, most often atrial tachycardia, in a cohort already recognized to carry elevated sudden death risk (115). In Ehlers-Danlos syndrome and hypermobility spectrum disorders, implantable loop recorders have also helped distinguish sinus tachycardia related to autonomic dysfunction from true arrhythmias, which in turn influenced management and, in rare cases, led to ablation or ICD placement (114). These findings support a targeted approach to rhythm surveillance, particularly in patients with symptoms, suggestive imaging findings, family history, or known higher-risk syndromic features.

### 5.3 Genotype-informed surveillance

Genotype is also likely to become increasingly relevant in arrhythmia surveillance, although its role is still evolving. In heritable thoracic aortic disease cohorts, arrhythmias and impaired myocardial function have been reported most often among patients with *FBN1* and TGF-β pathway variants, supporting the idea that some genotypes carry a more intrinsically arrhythmogenic myocardial phenotype (25). Among these, *SMAD3* appears particularly important, with reports of malignant ventricular arrhythmias and sudden cardiac death even in the absence of advanced structural disease (32, 33). These observations do not yet justify genotype alone as a management trigger, but they do suggest that certain variants should heighten clinical suspicion and may eventually help guide decisions about imaging, ambulatory monitoring, and follow-up intensity. At present, genotype is best viewed as a risk modifier that adds context rather than a stand-alone determinant of care (3).

### 5.4 Therapeutic approaches

Management of arrhythmias in CTDs requires balancing conventional rhythm strategies against the procedural and structural risks unique to connective tissue disease. In some settings, standard approaches remain appropriate, particularly for atrial arrhythmias or mitral valve-associated ventricular ectopy in patients without marked tissue fragility. In others, the connective tissue substrate itself meaningfully alters risk. This is especially evident in Ehlers-Danlos syndrome, where even routine ICD implantation has been associated with serious complications including myocardial tearing and vascular injury (112). Recent outcome data further demonstrate that invasive rhythm management presents distinct challenges in MFS and LDS. In MFS, among 41 patients undergoing rhythm-targeted atrial fibrillation ablation, 82.9% experienced recurrent atrial arrhythmia and 48.8% underwent multiple procedures; complications occurred in 28.4% of the overall 67-patient ablation cohort (120). In LDS, reported electrophysiologic interventions have included ablation, pacemaker implantation, and implantable cardioverter-defibrillator placement, with complications involving bleeding, cardiac perforation, thrombosis, infection, and lead failure in the available cohort (110). Postoperative data additionally emphasize that atrial arrhythmias are common after valve or aortic intervention in LDS (104). These findings do not preclude invasive treatment but support careful patient selection, procedural planning, and vigilant longitudinal follow-up. Accordingly, management should remain individualized and multidisciplinary, with the expected rhythm benefit weighed against recurrence risk and tissue-level procedural hazards.

### 5.5 Future research directions

Several next steps are likely to move the field forward. Prospective registries that combine genotype, advanced imaging, and longitudinal rhythm monitoring would help define the true prevalence and spectrum of arrhythmias across connective tissue disorders and clarify whether markers such as MAD or diffuse fibrosis consistently identify higher-risk patients. More data are also needed on peri-procedural outcomes, particularly in syndromes where vascular or myocardial fragility may complicate device implantation, ablation, or anticoagulation strategies. On the mechanistic side, pathways related to fibrosis and mechanotransduction, including SPARC-mediated collagen deposition and HIF-1*α* signaling, remain of interest as possible future therapeutic targets, although these ideas are still investigational (9, 86). For now, the most practical goal is not disease-specific antiarrhythmic therapy, but more informed surveillance and better recognition of which CTD patients may carry clinically meaningful electrical risk.

### 5.6 Limitations of the available evidence

The clinical evidence summarized in Table 1 is heterogeneous and remains dominated by retrospective referral cohorts, imaging studies, postoperative series, symptom-selected monitoring populations, administrative database analyses, and case reports. Several estimates were derived from patients selected because of non-aortic cardiac disease, surgery, ablation, or prolonged rhythm monitoring rather than from unselected CTD populations. Rhythm surveillance and outcome definitions were not standardized, control groups were frequently absent, and administrative studies are vulnerable to coding error, repeat hospitalizations, and residual confounding. The timing of rhythm diagnoses relative to aortic or valvular surgery was also inconsistently available, limiting separation of intrinsic arrhythmogenic substrate from postoperative remodeling and cumulative operative exposure. Formally published conference abstracts provide useful comparative data but offer less methodological detail than full-length reports. These limitations, together with potential publication bias toward unusual or severe presentations, prevent direct comparison of reported frequencies and preclude reliable estimation of population-level arrhythmia prevalence across CTDs.

The mechanistic evidence is similarly uneven. Many proposed relationships among CTD genes, fibrosis, mechanotransduction, ion-channel remodeling, and arrhythmia are supported by experimental models or analogy to inherited cardiomyopathies rather than direct genotype-to-arrhythmia studies in CTD cohorts. The evolving distinction between true and pseudo-MAD further limits comparisons among studies using different imaging definitions and cardiac phases. Accordingly, the available literature supports greater clinical awareness and individualized evaluation but does not establish causal pathways, validated risk thresholds, or universal surveillance and treatment strategies.

## 6 Conclusion

Connective tissue disorders have long been defined by their vascular complications, yet accumulating evidence shows that they can also predispose to clinically significant arrhythmias. Across Marfan, Loeys-Dietz, Ehlers-Danlos, and rarer syndromes, a recurring theme emerges: structural abnormalities such as mitral annular disjunction, diffuse fibrosis, and extracellular matrix fragility may create substrates for atrial and ventricular arrhythmias, sometimes independent of overt valvular or aortic disease.

At a mechanistic level, CTDs exemplify a continuum in which extracellular matrix disruption drives fibrosis, geometric remodeling, and altered mechanotransduction, ultimately culminating in electrical instability. Clinical studies, though limited, demonstrate arrhythmic events ranging from atrial fibrillation to malignant ventricular tachycardia, with risk often amplified by postoperative remodeling and tissue fragility.

These insights underscore the need to broaden the lens through which CTDs are viewed from disorders that are often narrowly defined by vascular risk to systemic syndromes with arrhythmogenic potential. Advanced imaging, ambulatory monitoring, and consideration of genetic context may support earlier recognition and targeted evaluation, but the evidence remains preliminary. Until prospective registries and translational trials clarify the true prevalence, mechanisms, and optimal management of arrhythmias in CTDs, clinical decisions must remain individualized, multidisciplinary, and cautious. Recognizing CTDs as dual vascular and electrical disorders provides a new framework for patient care and highlights a pressing agenda for future research.

## References

[1] Basso C Perazzolo Marra M Rizzo S De Lazzari M Giorgi B Cipriani A. Arrhythmic mitral valve prolapse and sudden cardiac death. Circulation. (2015) 132(7):556–66. 10.1161/CIRCULATIONAHA.115.016291 26160859

[2] Essayagh B Sabbag A El-Am E Cavalcante JL Michelena HI Enriquez-Sarano M. Arrhythmic mitral valve prolapse and mitral annular disjunction: pathophysiology, risk stratification, and management. Eur Heart J. (2023) 44(33):3121–35. 10.1093/eurheartj/ehad491 37561995

[3] Yagyu T Noguchi T Asano Y Ida K Ogata S Nishimura K. Association between genetic diagnosis and clinical outcomes in patients with heritable thoracic aortic disease. J Am Heart Assoc. (2023) 12(8):e028625. 10.1161/JAHA.122.028625 37042257 PMC10227281

[4] Karakasis P Theofilis P Vlachakis PK Korantzopoulos P Patoulias D Antoniadis AP. Atrial fibrosis in atrial fibrillation: mechanistic insights, diagnostic challenges, and emerging therapeutic targets. Int J Mol Sci. (2025) 26(1):209. 10.3390/ijms26010209 PMC11720255 39796066

[5] Chen C Chen P Yu W. TGF-beta-driven atrial fibrosis in atrial fibrillation: from mechanistic insights to targeted therapies. Aging Dis. (2025) 17:2509–28. 10.14336/AD.2025.0564 40768638 PMC13437098

[6] Robertson EN Bannon PG Jeremy RW. Long-term outcomes in heritable thoracic aortic disease. Front Cardiovasc Med. (2022) 9:1009947. 10.3389/fcvm.2022.1009947 36312254 PMC9606219

[7] Poe A Martinez Yus M Wang H Santhanam L. Lysyl oxidase like-2 in fibrosis and cardiovascular disease. Am J Physiol Cell Physiol. (2023) 325(3):C694–707. 10.1152/ajpcell.00176.2023 37458436 PMC10635644

[8] Trombetta-Esilva J Bradshaw AD. The function of SPARC as a mediator of fibrosis. Open Rheumatol J. (2012) 6:146–55. 10.2174/1874312901206010146 22802913 PMC3395844

[9] Zhou F Zhou JB Wei TP Wu D Wang RX. The role of HIF-1alpha in atrial fibrillation: recent advances and therapeutic potentials. Rev Cardiovasc Med. (2025) 26(2):26787. 10.31083/RCM26787 40026494 PMC11868874

[10] Sabbag A Essayagh B Barrera JDR Basso C Berni A Cosyns B. EHRA Expert consensus statement on arrhythmic mitral valve prolapse and mitral annular disjunction complex in collaboration with the ESC council on valvular heart disease and the European association of cardiovascular imaging endorsed by the heart rhythm society, the Asia Pacific heart rhythm society, and the Latin American heart rhythm society. Europace. (2022) 24(12):1981–2003. 10.1093/europace/euac125 35951656 PMC11636573

[11] Fiore G Rizza V Ingallina G Ancona F Stella S Biondi F. Prevalence of diastolic and systolic mitral annular disjunction in patients with mitral valve prolapse. J Am Soc Echocardiogr. (2025) 38(1):1–11. 10.1016/j.echo.2024.10.004 39442734

[12] Motwani M Venetucci L. Mitral annular disjunction arrhythmia syndrome in Marfan syndrome. Eur Heart J Case Rep. (2020) 4(5):1–2. 10.1093/ehjcr/ytaa306 PMC7780496 33426461

[13] Toomer KA Yu M Fulmer D Guo L Moore KS Moore R. Primary cilia defects causing mitral valve prolapse. Sci Transl Med. (2019) 11(493):eaax0290. 10.1126/scitranslmed.aax0290 31118289 PMC7331025

[14] Durst R Sauls K Peal DS Devlaming A Toomer K Leyne M. Mutations in DCHS1 cause mitral valve prolapse. Nature. (2015) 525(7567):109–13. 10.1038/nature14670 26258302 PMC4720389

[15] Garg V Muth AN Ransom JF Schluterman MK Barnes R King IN. Mutations in NOTCH1 cause aortic valve disease. Nature. (2005) 437(7056):270–4. 10.1038/nature03940 16025100

[16] Kyndt F Gueffet J-P Probst V Jaafar P Legendre A Le Bouffant F. Mutations in the gene encoding filamin A as a cause for familial cardiac valvular dystrophy. Circulation. (2007) 115(1):40–9. 10.1161/CIRCULATIONAHA.106.622621 17190868

[17] Ortiz-Genga MF Cuenca S Dal Ferro M Zorio E Salgado-Aranda R Climent V. Truncating FLNC mutations are associated with high-risk dilated and arrhythmogenic cardiomyopathies. J Am Coll Cardiol. (2016) 68(22):2440–51. 10.1016/j.jacc.2016.09.927 27908349

[18] Gigli M Merlo M Graw SL Barbati G Rowland TJ Slavov DB. Genetic risk of arrhythmic phenotypes in patients with dilated cardiomyopathy. J Am Coll Cardiol. (2019) 74(11):1480–90. 10.1016/j.jacc.2019.06.072 31514951 PMC6750731

[19] Zhang JM Au DT Sawada H Franklin MK Moorleghen JJ Howatt DA. LRP1 Protects against excessive superior mesenteric artery remodeling by modulating angiotensin II-mediated signaling. JCI Insight. (2023) 8(2):e164751. 10.1172/jci.insight.164751 36472907 PMC9977308

[20] Silva AC Pereira C Fonseca A Pinto-do OP Nascimento DS. Bearing my heart: the role of extracellular matrix on cardiac development, homeostasis, and injury response. Front Cell Dev Biol. (2020) 8:621644. 10.3389/fcell.2020.621644 33511134 PMC7835513

[21] Sykora M Szeiffova Bacova B Andelova K Egan Benova T Martiskova A Kurahara L-H. Connexin43, a promising target to reduce cardiac arrhythmia burden in pulmonary arterial hypertension. Int J Mol Sci. (2024) 25(6):3275. 10.3390/ijms25063275 38542257 PMC10970272

[22] Thiene G Basso C Pilichou K Bueno Marinas M. Desmosomal arrhythmogenic cardiomyopathy: the story telling of a genetically determined heart muscle disease. Biomedicines. (2023) 11(7):2018. 10.3390/biomedicines11072018 37509658 PMC10377062

[23] Brandao M Bariani R Rigato I Bauce B. Desmoplakin cardiomyopathy: comprehensive review of an increasingly recognized entity. J Clin Med. (2023) 12(7):2660. 10.3390/jcm12072660 37048743 PMC10095332

[24] Dreher L Abdul Nabi H Vandolah H Brennan S Bcharah G Bcharah H. The expanding genetic architecture of arteriopathies: from canonical TAAD genes to emerging connective tissue and signaling pathways. Med Sci (Basel). (2025) 13(3):155. 10.3390/medsci13030155 40981152 PMC12452550

[25] Demolder A Bianco L Caruana M Cervi E Evangelista A Jondeau G. Arrhythmia and impaired myocardial function in heritable thoracic aortic disease: an international retrospective cohort study. Eur J Med Genet. (2022) 65(6):104503. 10.1016/j.ejmg.2022.104503 35427808

[26] Sygitowicz G Maciejak-Jastrzebska A Sitkiewicz D. A review of the molecular mechanisms underlying cardiac fibrosis and atrial fibrillation. J Clin Med. (2021) 10(19):4430. 10.3390/jcm10194430 34640448 PMC8509789

[27] Asokan KL Landes JR Renders W Muiño Mosquera L De Backer J Jantzen DW. Mitral annular disjunction in heritable thoracic aortic disease: insights from the Montalcino Aortic Consortium. J Am Heart Assoc. (2024) 13(21):e036274. 10.1161/JAHA.124.036274 39424426 PMC11935710

[28] MacCarrick G Black JH Bowdin S El-Hamamsy I Frischmeyer-Guerrerio PA Guerrerio AL. Loeys-Dietz syndrome: a primer for diagnosis and management. Genet Med. (2014) 16(8):576–87. 10.1038/gim.2014.11 24577266 PMC4131122

[29] Loeys BL Schwarze U Holm T Callewaert BL Thomas GH Pannu H. Aneurysm syndromes caused by mutations in the TGF-beta receptor. N Engl J Med. (2006) 355(8):788–98. 10.1056/NEJMoa055695 16928994

[30] Kunamalla A Ng J Parini V Yoo S McGee KA Tomson TT. Constitutive expression of a dominant-negative TGF-beta type II receptor in the posterior left atrium leads to beneficial remodeling of atrial fibrillation substrate. Circ Res. (2016) 119(1):69–82. 10.1161/CIRCRESAHA.115.307878 27217399 PMC4920729

[31] Saadat S Noureddini M Mahjoubin-Tehran M Nazemi S Shojaie L Aschner M. Pivotal role of TGF-beta/smad signaling in cardiac fibrosis: non-coding RNAs as effectual players. Front Cardiovasc Med. (2020) 7:588347. 10.3389/fcvm.2020.588347 33569393 PMC7868343

[32] Backer J Braverman AC. Heart failure and sudden cardiac death in heritable thoracic aortic disease caused by pathogenic variants in the SMAD3 gene. Mol Genet Genomic Med. (2018) 6(4):648–52. 10.1002/mgg3.396 29717556 PMC6081213

[33] van De Laar IMBH Oldenburg RA Pals G Roos-Hesselink JW De Graaf BM Verhagen JMA. Mutations in SMAD3 cause a syndromic form of aortic aneurysms and dissections with early-onset osteoarthritis. Nat Genet. (2011) 43(2):121–6. 10.1038/ng.744 21217753

[34] Burkitt Wright EMM Spencer HL Daly SB Manson FDC Zeef LAH Urquhart J. Mutations in PRDM5 in brittle cornea syndrome identify a pathway regulating extracellular matrix development and maintenance. Am J Hum Genet. (2011) 88(6):767–77. 10.1016/j.ajhg.2011.05.007 21664999 PMC3113239

[35] Yang S Li Z. FBN2 Pathogenic variants in congenital contractural arachnodactyly with severe cardiovascular manifestations. Connect Tissue Res. (2024) 65(3):214–25. 10.1080/03008207.2024.2340004 38602424

[36] Zhou N Zhao Q Li R Cheng R Wu Q Cheng J. Mutation in mitral valve prolapse susceptible gene DCHS1 causes familial mitral annular disjunction. J Med Genet. (2024) 61(2):125–31. 10.1136/jmg-2023-109278 37399314

[37] Beaufort N Scharrer E Kremmer E Lux V Ehrmann M Huber R. Cerebral small vessel disease-related protease HtrA1 processes latent TGF-beta binding protein 1 and facilitates TGF-beta signaling. Proc Natl Acad Sci U S A. (2014) 111(46):16496–501. 10.1073/pnas.1418087111 25369932 PMC4246310

[38] Fasano A Formichi P Taglia I Bianchi S Di Donato I Battisti C. HTRA1 Expression profile and activity on TGF-beta signaling in HTRA1 mutation carriers. J Cell Physiol. (2020) 235(10):7120–7. 10.1002/jcp.29609 32017060

[39] Le Tourneau T Mérot J Rimbert A Le Scouarnec S Probst V Le Marec H. Genetics of syndromic and non-syndromic mitral valve prolapse. Heart. (2018) 104(12):978–84. 10.1136/heartjnl-2017-312420 29352010 PMC6168077

[40] Guo L Beck T Fulmer D Ramos-Ortiz S Glover J Wang C. DZIP1 Regulates mammalian cardiac valve development through a Cby1-beta-catenin mechanism. Dev Dyn. (2021) 250(10):1432–49. 10.1002/dvdy.342 33811421 PMC8518365

[41] Le Tourneau T Le Scouarnec S Cueff C Bernstein D Aalberts JJJ Lecointe S. New insights into mitral valve dystrophy: a Filamin-A genotype-phenotype and outcome study. Eur Heart J. (2018) 39(15):1269–77. 10.1093/eurheartj/ehx505 29020406 PMC5905589

[42] Toomer K Sauls K Fulmer D Guo L Moore K Glover J. Filamin-A as a balance between erk/smad activities during cardiac valve development. Anat Rec (Hoboken). (2019) 302(1):117–24. 10.1002/ar.23911 30288957 PMC6312478

[43] Dina C Bouatia-Naji N Tucker N Delling FN Toomer K Durst R. Genetic association analyses highlight biological pathways underlying mitral valve prolapse. Nat Genet. (2015) 47(10):1206–11. 10.1038/ng.3383 26301497 PMC4773907

[44] Wang Y Wu B Chamberlain AA Lui W Koirala P Susztak K. Endocardial to myocardial notch-wnt-bmp axis regulates early heart valve development. PLoS One. (2013) 8(4):e60244. 10.1371/journal.pone.0060244 23560082 PMC3613384

[45] Rath N Wang Z Lu MM Morrisey EE. LMCD1/Dyxin Is a novel transcriptional cofactor that restricts GATA6 function by inhibiting DNA binding. Mol Cell Biol. (2005) 25(20):8864–73. 10.1128/MCB.25.20.8864-8873.2005 16199866 PMC1265795

[46] Cameron JN Kadhim KI Kamsani SHB Han H-C Farouque O Sanders P. Arrhythmogenic mitral valve prolapse: can we risk stratify and prevent sudden cardiac death? Arrhythm Electrophysiol Rev. (2024) 13:e11. 10.15420/aer.2023.26 39145277 PMC11322952

[47] Arrigo AB Zhu W Williams KA Guzman-Moreno C Lo C Lin JI. Contribution of LRP1 in human congenital heart disease correlates with its roles in the outflow tract and atrioventricular cushion development. Genes (Basel). (2023) 14(4):947. 10.3390/genes14040947 37107705 PMC10137934

[48] Strickland DK Au DT Cunfer P Muratoglu SC. Low-density lipoprotein receptor-related protein-1: role in the regulation of vascular integrity. Arterioscler Thromb Vasc Biol. (2014) 34(3):487–98. 10.1161/ATVBAHA.113.301924 24504736 PMC4304649

[49] Wang Y Li C Shi L Chen X Cui C Huang J. Integrin beta1D deficiency-mediated RyR2 dysfunction contributes to catecholamine-sensitive ventricular tachycardia in arrhythmogenic right ventricular cardiomyopathy. Circulation. (2020) 141(18):1477–93. 10.1161/CIRCULATIONAHA.119.043504 32122157 PMC7200284

[50] Tangney JR Chuang JS Janssen MS Krishnamurthy A Liao P Hoshijima M. Novel role for vinculin in ventricular myocyte mechanics and dysfunction. Biophys J. (2013) 104(7):1623–33. 10.1016/j.bpj.2013.02.021 23561539 PMC3617425

[51] Pannu H Tran-Fadulu V Papke CL Scherer S Liu Y Presley C. MYH11 Mutations result in a distinct vascular pathology driven by insulin-like growth factor 1 and angiotensin II. Hum Mol Genet. (2007) 16(20):2453–62. 10.1093/hmg/ddm201 17666408 PMC2905218

[52] Zhu S Ye L Bennett S Xu H He D Xu J. Molecular structure and function of microfibrillar-associated proteins in skeletal and metabolic disorders and cancers. J Cell Physiol. (2021) 236(1):41–8. 10.1002/jcp.29893 32572962

[53] Hannuksela M Stattin E-L Klar J Ameur A Johansson B Sörensen K. A novel variant in MYLK causes thoracic aortic dissections: genotypic and phenotypic description. BMC Med Genet. (2016) 17(1):61. 10.1186/s12881-016-0326-y 27586135 PMC5008005

[54] Wallace SE Regalado ES Gong L Janda AL Guo D- Russo CF. MYLK Pathogenic variants aortic disease presentation, pregnancy risk, and characterization of pathogenic missense variants. Genet Med. (2019) 21(1):144–51. 10.1038/s41436-018-0038-0 29925964 PMC6400320

[55] Norton EL Gordon D Yang B. Managing the aorta in patients with a PRKG1 mutation. J Thorac Cardiovasc Surg. (2019) 157(4):e107–9. 10.1016/j.jtcvs.2018.09.097 30415903 PMC7068642

[56] Numata G Takimoto E. Cyclic GMP and PKG signaling in heart failure. Front Pharmacol. (2022) 13:792798. 10.3389/fphar.2022.792798 35479330 PMC9036358

[57] Choudhury R Revenco V Darciuc R. Ehlers-Danlos syndrome. BMJ Case Rep. (2009) 2009:bcr05.2009.1850. 10.1136/bcr.05.2009.1850 21754955 PMC3028600

[58] Malfait F Wenstrup RJ De Paepe A. Clinical and genetic aspects of ehlers-danlos syndrome, classic type. Genet Med. (2010) 12(10):597–605. 10.1097/GIM.0b013e3181eed412 20847697

[59] Henneton P Legrand A Giunta C Frank M. Arterial fragility in kyphoscoliotic ehlers-danlos syndrome. BMJ Case Rep. (2018) 2018:bcr2018224423. 10.1136/bcr-2018-224423 29982180 PMC6040561

[60] Li H Zhu X Cao X Lu Y Zhou J Zhang X. Single-cell analysis reveals lysyl oxidase (Lox)(+) fibroblast subset involved in cardiac fibrosis of diabetic mice. J Adv Res. (2023) 54:223–37. 10.1016/j.jare.2023.01.018 36706988 PMC10703720

[61] Rodriguez C Martinez-Gonzalez J. The role of lysyl oxidase enzymes in cardiac function and remodeling. Cells. (2019) 8(12):1483. 10.3390/cells8121483 31766500 PMC6953057

[62] Cocciolone AJ Hawes JZ Staiculescu MC Johnson EO Murshed M Wagenseil JE. Elastin, arterial mechanics, and cardiovascular disease. Am J Physiol Heart Circ Physiol. (2018) 315(2):H189–205. 10.1152/ajpheart.00087.2018 29631368 PMC6139627

[63] Lage JGB Bortolotto AL Scanavacca MI Bortolotto LA Darrieux F. Arterial stiffness and atrial fibrillation: a review. Clinics (Sao Paulo). (2022) 77:100014. 10.1016/j.clinsp.2022.100014 35248986 PMC8903742

[64] Gharesouran J Hosseinzadeh H Ghafouri-Fard S Jabbari Moghadam Y Ahmadian Heris J Jafari-Rouhi AH. New insight into clinical heterogeneity and inheritance diversity of FBLN5-related cutis laxa. Orphanet J Rare Dis. (2021) 16(1):51. 10.1186/s13023-021-01696-6 33509220 PMC7845118

[65] Hucthagowder V Sausgruber N Kim KH Angle B Marmorstein LY Urban Z. Fibulin-4: a novel gene for an autosomal recessive cutis laxa syndrome. Am J Hum Genet. (2006) 78(6):1075–80. 10.1086/504304 16685658 PMC1474103

[66] Ramachandra AB Mikush N Sauler M Humphrey JD Manning EP. Compromised cardiopulmonary function in fibulin-5 deficient mice. J Biomech Eng. (2022) 144(8):081008. 10.1115/1.4053873 35171214 PMC8990734

[67] Zhang Y Marmorstein LY. Focus on molecules: fibulin-3 (EFEMP1). Exp Eye Res. (2010) 90(3):374–5. 10.1016/j.exer.2009.09.018 19799900 PMC2896546

[68] Zhi K Yin R Guo H Qu L. PUM2 Regulates the formation of thoracic aortic dissection through EFEMP1. Exp Cell Res. (2023) 427(2):113602. 10.1016/j.yexcr.2023.113602 37062520

[69] Demolder A Timmermans F Duytschaever M Muino-Mosquera L De Backer J. Association of mitral annular disjunction with cardiovascular outcomes among patients with Marfan syndrome. JAMA Cardiol. (2021) 6(10):1177–86. 10.1001/jamacardio.2021.2312 34232254 PMC8506229

[70] Barbier M Gross M-S Aubart M Hanna N Kessler K Guo D-C. MFAP5 loss-of-function mutations underscore the involvement of matrix alteration in the pathogenesis of familial thoracic aortic aneurysms and dissections. Am J Hum Genet. (2014) 95(6):736–43. 10.1016/j.ajhg.2014.10.018 25434006 PMC4259978

[71] Jin Y Li T Wu S Liu Z Li Y. MFAP5 variant-induced multiple giant thoracic aortic aneurysm. Cardiol Young. (2024) 34(1):212–7. 10.1017/S1047951123004122 38031457

[72] Dagoneau N Benoist-Lasselin C Huber C Faivre L Mégarbané A Alswaid A. ADAMTS10 Mutations in autosomal recessive Weill-Marchesani syndrome. Am J Hum Genet. (2004) 75(5):801–6. 10.1086/425231 15368195 PMC1182109

[73] Rypdal KB Apte SS Lunde IG. Emerging roles for the ADAMTS-like family of matricellular proteins in cardiovascular disease through regulation of the extracellular microenvironment. Mol Biol Rep. (2024) 51(1):280. 10.1007/s11033-024-09255-5 38324186 PMC10850197

[74] Wang Z Li W Chen S Tang XX. Role of ADAM and ADAMTS proteases in pathological tissue remodeling. Cell Death Discov. (2023) 9(1):447. 10.1038/s41420-023-01744-z 38071234 PMC10710407

[75] Beetz N Rommel C Schnick T Neumann E Lother A Monroy-Ordonez EB. Ablation of biglycan attenuates cardiac hypertrophy and fibrosis after left ventricular pressure overload. J Mol Cell Cardiol. (2016) 101:145–55. 10.1016/j.yjmcc.2016.10.011 27789290

[76] Johnson BB Cosson M-V Tsansizi LI Holmes TL Gilmore T Hampton K. Perlecan (HSPG2) promotes structural, contractile, and metabolic development of human cardiomyocytes. Cell Rep. (2024) 43(1):113668. 10.1016/j.celrep.2023.113668 38198277

[77] Kaufman CS Butler MG. Mutation in TNXB gene causes moderate to severe ehlers-danlos syndrome. World J Med Genet. (2016) 6(2):17–21. 10.5496/wjmg.v6.i2.17 28344932 PMC5363719

[78] Nakajima M Mizumoto S Miyake N Kogawa R Iida A Ito H. Mutations in B3GALT6, which encodes a glycosaminoglycan linker region enzyme, cause a spectrum of skeletal and connective tissue disorders. Am J Hum Genet. (2013) 92(6):927–34. 10.1016/j.ajhg.2013.04.003 23664117 PMC3675233

[79] Ritelli M Dordoni C Cinquina V Venturini M Calzavara-Pinton P Colombi M. Expanding the clinical and mutational spectrum of B4GALT7-spondylodysplastic ehlers-danlos syndrome. Orphanet J Rare Dis. (2017) 12(1):153. 10.1186/s13023-017-0704-3 28882145 PMC5590203

[80] Van Damme T Pang X Guillemyn B Gulberti S Syx D De Rycke R. Biallelic B3GALT6 mutations cause spondylodysplastic Ehlers-Danlos syndrome. Hum Mol Genet. (2018) 27(20):3475–87. 10.1093/hmg/ddy234 29931299

[81] Chakravarti S Magnuson T Lass JH Jepsen KJ LaMantia C Carroll H. Lumican regulates collagen fibril assembly: skin fragility and corneal opacity in the absence of lumican. J Cell Biol. (1998) 141(5):1277–86. 10.1083/jcb.141.5.1277 9606218 PMC2137175

[82] Mohammadzadeh N Lunde IG Andenæs K Strand ME Aronsen JM Skrbic B. The extracellular matrix proteoglycan lumican improves survival and counteracts cardiac dilatation and failure in mice subjected to pressure overload. Sci Rep. (2019) 9(1):9206. 10.1038/s41598-019-45651-9 31235849 PMC6591256

[83] Shen Y Russo V Zeglinski MR Sellers SL Wu Z Oram C. Recombinant decorin fusion protein attenuates murine abdominal aortic aneurysm formation and rupture. Sci Rep. (2017) 7(1):15857. 10.1038/s41598-017-16194-8 29158532 PMC5696466

[84] Vanlerberghe R Colige A Malfait AM Syx D Malfait F. ADAMTS2: more than a procollagen N-proteinase. Genes Dis. (2025) 12(6):101686. 10.1016/j.gendis.2025.101686 40837410 PMC12362006

[85] Coucke PJ Willaert A Wessels MW Callewaert B Zoppi N De Backer J. Mutations in the facilitative glucose transporter GLUT10 alter angiogenesis and cause arterial tortuosity syndrome. Nat Genet. (2006) 38(4):452–7. 10.1038/ng1764 16550171

[86] Riley HJ Kelly RR Van Laer AO Neff LS Dasgupta S Baicu CF. SPARC Production by bone marrow-derived cells contributes to myocardial fibrosis in pressure overload. Am J Physiol Heart Circ Physiol. (2021) 320(2):H604–12. 10.1152/ajpheart.00552.2020 33306449 PMC8082795

[87] Altmann HM Tester DJ Will ML Middha S Evans JM Eckloff BW. Homozygous/compound heterozygous triadin mutations associated with autosomal-recessive long-QT syndrome and pediatric sudden cardiac arrest: elucidation of the triadin knockout syndrome. Circulation. (2015) 131(23):2051–60. 10.1161/CIRCULATIONAHA.115.015397 25922419

[88] Baig SM Koschak A Lieb A Gebhart M Dafinger C Nürnberg G. Loss of Ca(v)1.3 (CACNA1D) function in a human channelopathy with bradycardia and congenital deafness. Nat Neurosci. (2011) 14(1):77–84. 10.1038/nn.2694 21131953

[89] Bergeman AT Wilde AAM Van Der Werf C. Catecholaminergic polymorphic ventricular tachycardia: a review of therapeutic strategies. Card Electrophysiol Clin. (2023) 15(3):293–305. 10.1016/j.ccep.2023.04.002 37558300

[90] Jiang C Zhang Y. Current updates on arrhythmia within timothy syndrome: genetics, mechanisms and therapeutics. Expert Rev Mol Med. (2023) 25:e17. 10.1017/erm.2023.11 37132248 PMC10407238

[91] Kapplinger JD Pundi KN Larson NB Callis TE Tester DJ Bikker H. Yield of the RYR2 genetic test in suspected catecholaminergic polymorphic ventricular tachycardia and implications for test interpretation. Circ Genom Precis Med. (2018) 11(2):e001424. 10.1161/CIRCGEN.116.001424 29453246 PMC6364978

[92] Li W Yin L Shen C Hu K Ge J Sun A. SCN5A Variants: association with cardiac disorders. Front Physiol. (2018) 9:1372. 10.3389/fphys.2018.01372 30364184 PMC6191725

[93] Mohler PJ Le Scouarnec S Denjoy I Lowe JS Guicheney P Caron L. Defining the cellular phenotype of “ankyrin-B syndrome” variants: human ANK2 variants associated with clinical phenotypes display a spectrum of activities in cardiomyocytes. Circulation. (2007) 115(4):432–41. 10.1161/CIRCULATIONAHA.106.656512 17242276

[94] Mohler PJ Schott J-J Gramolini AO Dilly KW Guatimosim S Dubell WH. Ankyrin-B mutation causes type 4 long-QT cardiac arrhythmia and sudden cardiac death. Nature. (2003) 421(6923):634–9. 10.1038/nature01335 12571597

[95] Mohler PJ Splawski I Napolitano C Bottelli G Sharpe L Timothy K. A cardiac arrhythmia syndrome caused by loss of ankyrin-B function. Proc Natl Acad Sci U S A. (2004) 101(24):9137–42. 10.1073/pnas.0402546101 15178757 PMC428486

[96] Ozawa J Ohno S Saito H Saitoh A Matsuura H Horie M. A novel CACNA1C mutation identified in a patient with timothy syndrome without syndactyly exerts both marked loss- and gain-of-function effects. HeartRhythm Case Rep. (2018) 4(7):273–7. 10.1016/j.hrcr.2018.03.003 30023270 PMC6050422

[97] Postma AV Denjoy I Hoorntje TM Lupoglazoff J-M Da Costa A Sebillon P. Absence of calsequestrin 2 causes severe forms of catecholaminergic polymorphic ventricular tachycardia. Circ Res. (2002) 91(8):e21–6. 10.1161/01.res.0000038886.18992.6b 12386154

[98] Rivaud MR Delmar M Remme CA. Heritable arrhythmia syndromes associated with abnormal cardiac sodium channel function: ionic and non-ionic mechanisms. Cardiovasc Res. (2020) 116(9):1557–70. 10.1093/cvr/cvaa082 32251506 PMC7341171

[99] Fan W Sun X Yuan R Hou X Wan J Liao B. HCN4 And arrhythmias: insights into base mutations. Mutat Res Rev Mutat Res. (2025) 795:108534. 10.1016/j.mrrev.2025.108534 39922561

[100] Milanesi R Baruscotti M Gnecchi-Ruscone T DiFrancesco D. Familial sinus bradycardia associated with a mutation in the cardiac pacemaker channel. N Engl J Med. (2006) 354(2):151–7. 10.1056/NEJMoa052475 16407510

[101] Dreher L Abdul Nabi H VanDolah H Shamoun FE El Masry HZ. PO-03-098 distinct atrial arrhythmia profiles among heritable aortopathies: comparative analysis of Marfan, Loeys-Dietz, and vascular EDS. Heart Rhythm. (2026) 23(4 Suppl):S550. 10.1016/j.hrthm.2026.03.837

[102] Kanaan C Bcharah G Bcharah H Abdul Nabi H Ibrahim R Dreher L. Ventricular tachycardia in patients with vascular Ehlers-Danlos syndrome vs Marfan syndrome: an international database analysis. Eur Heart J. (2025) 46(Suppl 1):ehaf784.606. 10.1093/eurheartj/ehaf784.606

[103] Abdul Nabi H Dreher LA Kanaan C Mousa H Kandil O Crossley A. Cardiac surgical outcomes and predictors of redo sternotomy in patients with Marfan syndrome: a retrospective multicentre cohort study. Open Heart. (2026) 13(1):e004138. 10.1136/openhrt-2026-004138 42229999 PMC13239629

[104] Nabi HA Kumar S Dreher L Bcharah G Odeh N Alatout M. Postoperative outcomes and valvular involvement in Loeys-Dietz syndrome: a multicenter cohort study of surgical risk and complications. Am J Cardiol. (2025) 257:110–5. 10.1016/j.amjcard.2025.08.032 40848925

[105] Nabi HA Dreher L Raslan MA Ibrahim R Bcharah H Gassner R. 26-A-16150-ACC Prevalence and sex-based differences in cardiac arrhythmias among adults with nonsyndromic thoracic aortic disease. J Am Coll Cardiol. (2026) 87(13 Suppl):A54–5. 10.1016/j.jacc.2026.02.139

[106] Wafa SEI Chahal CAA Sawatari H Khanji MY Khan H Asatryan B. Frequency of arrhythmias and postural orthostatic tachycardia syndrome in patients with Marfan syndrome: a nationwide inpatient study. J Am Heart Assoc. (2022) 11(17):e024939. 10.1161/JAHA.121.024939 36000435 PMC9496423

[107] Demolder A Devos D De Backer J Muino-Mosquera L. Assessment of myocardial fibrosis in Marfan syndrome using cardiac magnetic resonance imaging. Mol Genet Genomic Med. (2024) 12(11):e70024. 10.1002/mgg3.70024 39548726 PMC11568239

[108] Sanchez Parra CA Vaughan RM Iturralde Chavez A Miyake CY Valdes SO Pignatelli RH. Mitral annular disjunction is associated with ventricular arrhythmias in children with Marfan syndrome. J Am Soc Echocardiogr. (2025) 38(6):525–6. 10.1016/j.echo.2025.02.014 40054756

[109] Dey S Cheikhali R Frishman WH Aronow WS. Genetic problems, diagnosis, and cardiovascular manifestations of Loeys-Dietz syndrome. Cardiol Rev. (2024) 32(6):513–8. 10.1097/CRD.0000000000000544 37126428

[110] Nabi HA Bcharah G Bcharah H Dreher L Raslan MA Youssef A. The burden of cardiac arrhythmias in Loeys-Dietz syndrome: prevalence, etiology, and therapeutic interventions. JACC Adv. (2025) 4(11 Pt 1):102269. 10.1016/j.jacadv.2025.102269 41135387 PMC12593561

[111] Sanchez Tijmes F Chan VSH Murphy J Hashem DAL Hanneman K Wald RM. Mitral annular disjunction on cardiac MRI: prevalence and association with disease severity in Loeys-Dietz syndrome. Int J Cardiol. (2023) 392:131276. 10.1016/j.ijcard.2023.131276 37598908

[112] Garg V Bersohn M Han JK. Arrhythmias and myocardial fragility in Ehlers-Danlos syndrome: complications after routine ICD placement. HeartRhythm Case Rep. (2018) 4(7):301–3. 10.1016/j.hrcr.2018.03.008 30023276 PMC6050467

[113] Slawinski G Wabich E Hawryszko M Danilowicz-Szymanowicz L Chevalier P. Therapeutic difficulties in a patient with Ehlers-Danlos syndrome and numerous symptomatic premature ventricular contractions-case report and literature review. Front Cardiovasc Med. (2023) 10:1171541. 10.3389/fcvm.2023.1171541 37502188 PMC10368949

[114] Tale E Robinson G Edward J Kaushal R Riley B Cohen TJ. The utility of the implantable loop recorder in patients with Ehlers-Danlos syndrome and hypermobility spectrum disorder. J Osteopath Med. (2026) 126(3):123–8. 10.1515/jom-2025-0036 40530951

[115] Deitch AM Giacone HM Chubb H Algaze CA Lechich KM Collins RT. Arrhythmias in Williams syndrome. Am J Cardiol. (2023) 195:91–7. 10.1016/j.amjcard.2023.03.004 37037070

[116] Verdonk SJE Storoni S Micha D Van Den Aardweg JG Versacci P Celli L. Is osteogenesis Imperfecta associated with cardiovascular abnormalities? A systematic review of the literature. Calcif Tissue Int. (2024) 114(3):210–21. 10.1007/s00223-023-01171-3 38243143 PMC10902066

[117] Dairi AS Shihata M Bogis AA Alrefai M Aluthman U Jamjoom A. The role of cardiovascular surgery in the management of a patient diagnosed with congenital cutis laxa syndrome complicated by multivalvular heart disease. Cureus. (2021) 13(11):e19359. 10.7759/cureus.19359 34925973 PMC8654068

[118] Marzin P Cormier-Daire V Tsilou E. Weill-Marchesani syndrome. In: Adam MP Bick S Mirzaa GM, editors. GeneReviews®. Seattle: University of Washington (1993–2026). Accessed December 10, 2020. 20301293

[119] Rodriguez-Capitan J Macias-Benitez M Conejo-Munoz L Cordero-Aguilar A Lopez-Salguero R Perez-Villardon B. Arterial tortuosity syndrome: a late and unexpected diagnosis and description of a novel likely pathogenic mutation. Rev Esp Cardiol (Engl Ed). (2020) 73(6):504–6. 10.1016/j.rec.2019.10.010 31786173

[120] Nabi HA Dreher L Bcharah H Kanaan C Ibrahim R Hatem C. Efficacy and safety of atrial fibrillation ablation in patients with Marfan syndrome: a multicenter analysis. Heart Rhythm O2. (2026) 7(6):1053–8. 10.1016/j.hroo.2026.03.022 42369794 PMC13307489

