# Supplementary material for: Connective tissue disorders and cardiac arrhythmias: genetic pathways, mechanistic bridges, and clinical implications
Source: Front Cardiovasc Med. 2026 Sep 3;13:1860012. doi: 10.3389/fcvm.2026.1860012 (PMC13581506; doi:10.3389/fcvm.2026.1860012)
Supplement: Supplementary file 1 [file Table1.docx]

Table 1. Connective-Tissue Genes and Arrhythmia Susceptibility: Mechanisms, Phenotypes, and Clinical Considerations

| **Gene** | **Protein** | **Function** | **Location** | **Pathway Association** | **Syndrome / Primary Vascular Risk** | **Arrhythmia Association (Evidence Level)** | **Imaging / Biomarker Correlation** | **Clinical Considerations** |
| --- | --- | --- | --- | --- | --- | --- | --- | --- |
| ***ADAMTS10*** | ADAMTS-10 (secreted metalloproteinase) | Regulates microfibril assembly and ECM turnover | 19p13.3 | Microfibrils / ECM remodeling | Weill–Marchesani spectrum (microfibril dysfunction) | Emerging — microfibril defects can alter valve/annular stiffness and lower thresholds for fibrosis-driven arrhythmias | May contribute to MVP/MAD-like mechanics and basal LV fibrosis | Monitor geometry/fibrosis for substrate rather than primary channelopathy |
| ***ADAMTS2*** | Procollagen N-propeptidase | Cleaves N-terminal propeptides of fibrillar procollagens → collagen fibril maturation | 5q35.3 | Collagen processing / fibrillogenesis | Dermatosparaxis EDS; tissue/vascular fragility | Plausible — abnormal collagen fibrils promote patchy fibrosis and conduction heterogeneity | Diffuse/focal scar patterns on CMR may underlie AF/VT substrate | Procedural fragility can complicate ablation/ICD |
| ***B3GALT6*** | β-1,3-galactosyltransferase 6 (linker-region enzyme) | Builds proteoglycan linker tetrasaccharide; essential for GAG biosynthesis | 1p36.33 | Proteoglycans / linkeropathies | Spondylodysplastic EDS; vascular involvement | Emerging — impaired proteoglycan biosynthesis → ECM disorganization, fibrosis, conduction heterogeneity | Diffuse fibrosis on CMR; substrate accentuated with MVP/MAD | Substrate-oriented rhythm surveillance; procedural fragility awareness |
| ***B4GALT7*** | β-1,4-galactosyltransferase 7 (linker-region enzyme) | Adds galactose in proteoglycan linker synthesis | 5q35.3 | Proteoglycans / linkeropathies | Spondylodysplastic EDS; connective-tissue fragility | Emerging — ECM disarray predisposes to fibrosis-driven atrial/ventricular substrates | Diffuse fibrosis; annular laxity context | Focus on fibrosis/MAD imaging to guide monitoring |
| ***BGN*** | Biglycan (small leucine-rich proteoglycan) | Regulates collagen fibrillogenesis; pro-fibrotic fibroblast activation | Xq28 | Proteoglycans / fibrosis amplifier | X-linked skeletal/ECM fragility phenotypes | Plausible — preclinical loss reduces fibrosis and improves conduction; substrate-level driver | Diffuse fibrosis / conduction heterogeneity | Mechanistic relevance is primarily preclinical; clinical assessment should be based on the observed structural and rhythm phenotype. |
| ***COL1A1*** | Collagen type I α1 chain | Principal fibrillar collagen; tensile strength of myocardium/valves | 17q21.33 | Collagens / ECM tensile matrix | CTD-adjacent matrix fragility (not classical EDS type) | Plausible — excess/abnormal collagen deposition → interstitial fibrosis and anisotropy | Diffuse myocardial fibrosis on CMR; substrate for AF/VT | Anticipate scar-mediated arrhythmia despite preserved EF |
| ***COL1A2*** | Collagen type I α2 chain | Partners with COL1A1 in type I collagen fibrils | 7q21.3 | Collagens / ECM tensile matrix | CTD-adjacent matrix fragility | Plausible — promotes diffuse fibrosis and conduction heterogeneity (substrate-level) | Diffuse fibrosis; potential MVP/MAD interplay | Rhythm surveillance guided by scar burden rather than channelopathy |
| ***COL3A1*** | Type III collagen | Fibrillar collagen of vessels and myocardium | 2q32.2 | ECM tensile strength | Vascular EDS (arterial rupture, organ fragility) | Plausible (substrate-level) myocardial fragility → fibrosis/micro-scars; valve/annular laxity | Diffuse fibrosis; MVP/MAD substrate | Extreme tissue/vascular fragility → high interventional risk |
| ***COL5A1*** | Type V collagen α1 chain | Nucleation/regulation of type I collagen fibrillogenesis | 9q34.2–q34.3 | Collagen fibrillogenesis | Classical EDS | Plausible (substrate-level) myxomatous valves, annular laxity, diffuse fibrosis | MVP/MAD; diffuse fibrosis on CMR | Procedural fragility considerations |
| ***COL5A2*** | Type V collagen α2 chain | Partners with COL5A1 in regulating collagen fibrillogenesis | 2q32.2 | Collagen fibrillogenesis | Classical Ehlers–Danlos syndrome | Plausible (substrate-level) myxomatous valves, annular laxity, diffuse fibrosis | MVP/MAD; diffuse fibrosis risk | Tissue fragility complicates invasive rhythm care |
| ***DCHS1*** | Dachsous cadherin-related 1 (protocadherin) | Planar cell-polarity cue for directional VIC migration; leaflet morphogenesis; annular coupling | 11p15.4 | Planar cell polarity / cilia–mechanosensing | Familial myxomatous MVP often with MAD | Established for familial MVP/MAD; arrhythmic relevance is supported indirectly through the associated valve-annular phenotype, papillary muscle traction, and basal left ventricular fibrosis. | MAD on echo/CMR; basal inferolateral LV fibrosis in arrhythmogenic MVP | Consider assessment of MAD, ventricular fibrosis, and ectopy when the clinical valve phenotype is present, including when mitral regurgitation is not severe. |
| ***DCN*** | Decorin | Collagen-binding SLRP; sequesters TGF-β; anti-fibrotic modulator | 12q21.33 | Proteoglycans / fibrosis regulation | CTD-adjacent fibrosis regulation | Emerging — loss of decorin → diffuse fibrosis and conduction heterogeneity; supplementation attenuates arrhythmic remodeling | Reduced fibrosis with decorin augmentation in models (mechanistic) | Decorin remains an investigational antifibrotic pathway without established genotype-specific clinical implications. |
| ***DZIP1*** | DAZ-interacting zinc finger protein 1 | Centrosome/transition-zone protein required for primary cilia; restrains β-catenin in valve development | 13q32.1 | Cilia / planar cell polarity / Wnt–β-catenin | Monogenic MVP; atrioventricular regurgitation in zebrafish; myxomatous leaflet changes in mice | Established for monogenic MVP; its arrhythmic relevance is inferred from leaflet hypermobility, annular instability, and the resulting arrhythmogenic MVP phenotype. | MVP/MAD with basal LV fibrosis in high-risk phenotypes | Familial evaluation is appropriate for monogenic MVP; rhythm monitoring and imaging should be guided by the severity of the valve-annular phenotype and associated symptoms. |
| ***EFEMP1*** | Fibulin-3 (EFEMP1) | Elastic-fiber glycoprotein; supports elastin network integrity | 2p16.1 | Elastin/microfibril network; ECM transcriptional modifiers | Vascular/connective traits on GWAS; reduced expression in aneurysm tissue | Emerging — weakened elastic networks → afterload ↑, annular instability, fibrosis → conduction heterogeneity | Arterial stiffness; annular mechanics context | Evidence remains hypothesis-generating; clinical evaluation should be guided by the associated vascular, valvular, or myocardial phenotype. |
| ***EFEMP2*** | Fibulin-4 | Essential for elastogenesis and arterial wall integrity | 11q13.1 | Elastin–fibulin axis | Autosomal recessive cutis laxa (arterial tortuosity, aneurysms) | Emerging — elastic fiber failure → afterload ↑, annular mechanics impaired → fibrosis/conduction heterogeneity | Arterial stiffness; potential annular instability | Vascular tortuosity and fragility may limit invasive options. |
| ***ELN*** | Elastin | Core polymer of elastic fibers; regulates arterial compliance | 7q11.23 | Elastin–fibulin axis | Williams–Beuren syndrome, supravalvular aortic stenosis | Plausible (substrate-level) — arterial stiffness and valve anomalies linked to AF risk and ventricular substrate via MVP/MAD | Increased arterial stiffness; frequent valve/annular abnormalities | Rhythm surveillance in the context of vascular/valvular disease |
| ***FBLN5*** | Fibulin-5 | Links tropoelastin to microfibrils during elastic fiber assembly | 14q32.12 | Elastin–fibulin axis | AR cutis laxa with widespread arterial disease | Emerging — impaired elastogenesis → wall-stress shift, fibrosis, Cx43 disorganization and slowed conduction in models | Arterial stiffness; potential gap-junction remodeling | Substrate surveillance (fibrosis/MVP-MAD) rather than primary channelopathy |
| ***FBN1*** | Fibrillin-1 | Scaffold for microfibrils; regulates latent TGF-β sequestration | 15q21.1 | Microfibrils / TGF-β modulation | Marfan syndrome (aorta + myxomatous valve disease) | Established — MVP/MAD strongly linked to VA/SCD in MFS; dual biochemical (fibrosis) and geometric (papillary traction/Purkinje) mechanisms | Basal LV fibrosis: MAD quantification predicts outcomes | Rhythm surveillance and CMR fibrosis assessment in MFS, especially with MAD |
| ***FBN2*** | Fibrillin 2 | Fetally expressed microfibril scaffold for elastin | 5q23.3 | TGF-β / SMAD signaling | Congenital contractural arachnodactyly (occasional aortic dilation, valve disease). | Emerging — human reports are mainly structural (aortic/mitral); arrhythmia not systematically described but mechanistically plausible via annular/valve remodeling and fibrosis. | Mitral valve abnormalities, occasional MVP/MAD, suspected fibrosis. | No dedicated arrhythmia surveillance guidelines; possible under-recognition in congenital cohorts. |
| ***FLNA*** | Filamin A | Actin cross-linker; focal-adhesion mechanosensor; transduces shear/stretch to signaling | Xq28 | Focal adhesion / integrin mechanotransduction | X-linked valvular dystrophy with severe multivalvular prolapse; shortened chordae; annular alterations | Established — strain-sensing failure → leaflet billowing, MAD, basal LV fibrosis; pedigrees show arrhythmogenic potential | MAD and basal fibrosis on CMR in aggressive MVP | Male-predominant severity; early rhythm surveillance and consideration of high-risk MVP pathways |
| ***HIF1A (TGF-β–responsive modifier)*** | Hypoxia-inducible factor-1α | Transcriptional integrator of hypoxia/mechanical stress; augments pro-fibrotic TGF-β signaling | 14q23.2 | Fibrosis signaling / mechanotransduction | CTD-adjacent signaling amplifier | Emerging — elevated HIF-1α correlates with atrial fibrosis and AF persistence | Atrial/ventricular fibrosis burden tracks risk | HIF-1α remains an investigational fibrosis pathway; no HIF1A-specific rhythm surveillance strategy has been established. |
| ***HSPG2*** | Perlecan | Basement-membrane proteoglycan involved in growth-factor presentation and myocyte–matrix coupling | 1p36.12 | Proteoglycans / mechanotransduction | Perlecan-related connective-tissue disorders; cardiovascular phenotype incompletely defined | Emerging — experimental data support a role in cardiomyocyte structural and electrical maturation, but direct human evidence linking HSPG2 variants to arrhythmias is limited. | No established arrhythmia-specific imaging marker; fibrosis or annular abnormalities may provide indirect phenotypic context | Clinical implications remain investigational; rhythm and imaging evaluation should be guided by the observed phenotype rather than genotype alone |
| ***HTRA1*** | HtrA serine peptidase 1 | ECM protease; modulates TGF-β activity and matrix turnover | 10q26.13 | ECM turnover / TGF-β modulation | Cerebral small-vessel disease; arterial remodeling | Emerging — heightened activity promotes fibrosis and conduction barriers | Fibrosis burden aligns with risk | Consider pro-fibrotic signaling control; invasive procedures as needed |
| ***ITGA7*** | Integrin α7 (laminin receptor) | ECM–cytoskeleton coupling; traction sensing at costameres | 12q13.2 | Focal adhesion / integrin mechanotransduction | CTD-adjacent mechanotransduction | Plausible — integrin signaling regulates RyR2 stability and fibroblast activation → fibrosis and Ca²⁺ instability | Fibrosis and MAD severity inform monitoring | Mechanistic amplifier—use substrate-guided surveillance |
| ***ITGB1*** | Integrin β1 (laminin/collagen receptor subunit) | Core β subunit for multiple integrins; stabilizes sarcolemma and Ca²⁺ microdomains | 10p11.22 | Focal adhesion / integrin mechanotransduction | CTD-adjacent mechanotransduction | Plausible — β1-integrin deficiency/maladaptation → RyR2 dysfunction, fibrosis, and catecholamine-sensitive VT mechanisms | Diffuse fibrosis and ectopy burden guide follow-up | Mechanistic evidence implicates calcium-handling instability, but no ITGB1-specific treatment or surveillance strategy has been established. |
| ***LMCD1*** | LIM and cysteine-rich domains 1 (Dyxin) | LIM-domain transcriptional co-factor; represses GATA6; interfaces with calcineurin/NFAT | 3p25.3 | Transcriptional modifier (GATA6 / NFAT) affecting valve patterning | Polygenic MVP risk locus (GWAS/functional target); developmental mis-patterning with posterior leaflet thickening | Emerging — modifier that plausibly promotes MAD-like mechanics and annular hypermobility | MVP/MAD spectrum; fibrosis may be subtle | Treat as risk modifier—use MAD/fibrosis imaging in phenotype-positive carriers |
| ***LOX*** | Lysyl oxidase | Catalyzes collagen/elastin cross-linking; stiffens ECM | 5q23.2 | Cross-linking enzymes / fibrosis | Fibrosis-driven cardiac/vascular remodeling | Plausible — experimental and broader cardiovascular evidence links LOX upregulation to myocardial fibrosis, conduction slowing, and reentry, although direct CTD-specific arrhythmia data are limited. | Diffuse fibrosis; conduction slowing | The LOX pathway remains an investigational antifibrotic target; current clinical relevance is limited to its contribution to the fibrotic substrate. |
| ***LOXL1*** | Lysyl oxidase-like 1 | Collagen/elastin cross-linking; increases ECM stiffness and fibrosis | 15q24.1 | ECM cross-linking / fibrosis | Not a classic monogenic HTAD gene; LOX-family upregulation linked to cardiac/vascular fibrosis | Emerging — fibrosis-driven AF/VT substrate; strong mechanistic evidence, limited direct human arrhythmia data | Diffuse interstitial fibrosis (CMR/LGE), conduction slowing (substrate-level) | Clinical implications remain investigational; fibrosis burden may be more informative than genotype for assessing the arrhythmic substrate. |
| ***LOXL2*** | Lysyl oxidase-like 2 | Collagen/elastin cross-linking; promotes fibrotic matrix remodeling | 8p21.2–p21.3 | ECM cross-linking / fibrosis | As above for LOX family; fibrosis amplification rather than primary HTAD | Emerging — fibrosis-linked arrhythmic substrate; human arrhythmia cohorts limited | Fibrosis burden on CMR; slowed conduction/reentry risk in fibrotic myocardium | Clinical implications remain investigational; imaging and rhythm assessment should be guided by evidence of myocardial fibrosis or electrical abnormalities. |
| ***LRP1*** | Low-density lipoprotein receptor-related protein 1 | Endocytic and signaling receptor; regulates TGF-β trafficking, integrin-cytoskeletal signaling, and Ca²⁺ homeostasis | 12q13.3 | TGF-β / SMAD signaling | Associated with aneurysm biology, it contributes to TAAD via ECM and signaling regulation. | Plausible — integrates TGF-β trafficking with Ca²⁺ handling and cytoskeletal responses; experimental work links dysfunction to abnormal conduction, but human arrhythmia cohorts limited. | Experimental data support abnormal Ca²⁺ handling; fibrosis and conduction changes inferred. | LRP1 is best regarded as a mechanistic modifier; direct clinical arrhythmia implications have not been validated. |
| ***LUM*** | Lumican | Small leucine-rich proteoglycan; regulates collagen fibril diameter/spacing | 12q21.3–q22 | Proteoglycan ECM organization / fibrillogenesis | Not a classic vascular syndrome; modifier of myocardial stiffness/fibrosis | Plausible — experimental data support fibrosis-linked arrhythmogenic remodeling | Experimental data support altered matrix stiffness and fibrosis; no validated human imaging biomarker has been established. | Substrate-level risk; monitor fibrosis and arrhythmias even if systolic function is preserved. |
| ***MFAP2*** | Microfibril-associated glycoprotein-1 (MAGP-1) | Contributes to fibrillin microfibril assembly and growth-factor regulation | 1p36.13 | Microfibrils / TGF-β availability | CTD-adjacent microfibril dysfunction | Emerging — altered TGF-β/elastin homeostasis → myocardial fibrosis and annular remodeling | Diffuse fibrosis; MVP/MAD-adjacent mechanics | Monitor for substrate development in valve/annulus |
| ***MFAP5*** | Microfibril-associated glycoprotein-2 (MAGP-2) | Interacts with fibrillin-1 and integrins to organize elastin-containing microfibrils | 12p13.31 | Microfibrils / integrin signaling | Familial TAAD in some cohorts | Emerging — matrix dysregulation predisposes to interstitial fibrosis and annular remodeling (MVP/MAD context) | Fibrosis; valve-annulus geometry | Substrate-based rhythm surveillance |
| ***MYH11*** | Smooth muscle myosin heavy chain 11 | Vascular SMC contractile motor; regulates arterial tone | 16p13.11 | VSMC contractility / TAAD biology | Nonsyndromic HTAD; TAAD ± PDA (classic) | AF/ventricular ectopy (emerging) — likely secondary to geometry/afterload/fibrosis | Aortic dilation; valve involvement variably present | Vascular phenotype dominates; rhythm surveillance reasonable but not standardized |
| ***MYLK*** | Myosin light-chain kinase | Phosphorylates MLC to activate SMC contraction | 3q21.1 | VSMC contractility / TAAD biology | Early-onset/dissection-prone TAAD (often at smaller diameters) | AF/ventricular ectopy (emerging; indirect loading/fibrosis mechanisms) | Predominantly vascular; myocardial data limited | Intensive aortic surveillance; individualized rhythm monitoring |
| ***NOTCH1*** | Notch receptor 1 | Orchestrates endocardial-to-mesenchymal transition (EMT) and valve morphogenesis; restrains osteogenic signaling | 9q34.3 | Developmental patterning (Notch–Wnt–BMP axis) / EMT | Congenital aortic valve disease (incl. bicuspid aortic valve); pathway-level impact on valve/annulus geometry | Plausible — modifies leaflet/annular structure that amplifies MAD-driven arrhythmic risk; not a common monogenic MVP cause | Consider MAD and basal fibrosis assessment in structural valve disease | Structural focus; arrhythmia risk tracking in BAV/valve-dysplasia phenotypes |
| ***PLOD1*** | Lysyl hydroxylase-1 | Hydroxylates lysines in procollagen → enables fibril cross-linking | 1p36.22 | Collagen maturation / cross-linking | Kyphoscoliotic EDS (tissue/arterial fragility) | Plausible — compromised cross-linking → patchy fibrosis, anisotropic conduction | Diffuse/focal scar substrate | High procedural fragility risk |
| ***PRDM5*** | PR/SET domain 5 | Chromatin regulator of collagen/proteoglycan transcription; shifts ECM programs toward fibrosis | 4q27 | ECM transcriptional regulation | Brittle cornea syndrome (connective-tissue fragility) | Plausible — biology supports fibrosis/conduction heterogeneity as modifiers | No specific biomarker; anticipate diffuse interstitial fibrosis patterns when pathogenic | Lacks genotype-guided therapy emphasizes fibrosis surveillance and rhythm monitoring |
| ***PRKG1*** | cGMP-dependent protein kinase I (PKG I) | NO/cGMP effector; modulates VSMC relaxation, ECM signaling | 10q11.23 | cGMP signaling / TAAD biology | Early, often severe TAAD (gain-of-function variants described) | Arrhythmias (emerging; evidence limited and largely indirect) | Vascular phenotype dominates; myocardial fibrosis not systematically reported | Intensive aortic surveillance; arrhythmia monitoring individualized due to limited data |
| ***SLC2A10*** | GLUT10 | Glucose transporter; regulates redox tone and interfaces with TGF-β/ECM signaling | 20q13 (q13.12 region) | Redox/TGF-β–responsive ECM regulation | Arterial tortuosity syndrome (arterial tortuosity, aneurysm/dissection risk) | Emerging — hypothesis-level arrhythmia amplifier via fibrosis/oxidative stress | Aortic tortuosity on vascular imaging; myocardial fibrosis may be secondary | Manage vascular disease; consider rhythm surveillance in high-risk phenotypes |
| ***SMAD2*** | SMAD family member 2 | Intracellular mediator of TGF-β signaling; regulates gene transcription | 18q21.1 | TGF-β / SMAD signaling | Rare HTAD variants, not classic LDS subtype | Fibrosis-driven AF/VT in models; limited human data (emerging) | Experimental fibrosis, anisotropy; no consistent human imaging markers yet | Unclear penetrance; largely hypothesis-level in clinical practice |
| ***SMAD3*** | SMAD family member 3 | Intracellular mediator of TGF-β signaling; regulates gene transcription | 15q22.33 | TGF-β / SMAD signaling | Loeys–Dietz syndrome type 3 (aneurysm–osteoarthritis syndrome) | Malignant VT/VF, sudden cardiac death (established in multiple cohorts) | Myocardial fibrosis, MVP/MAD sometimes present | Arrhythmic risk disproportionate to vascular/valve disease; early rhythm monitoring and ICD discussion may be warranted |
| ***SMAD4*** | Mothers against decapentaplegic homolog 4 | Canonical intracellular mediator of TGF-β signaling; partners with SMAD2/3 to regulate transcription | 18q21.2 | TGF-β / SMAD signaling | Juvenile polyposis–hereditary hemorrhagic telangiectasia (JPS-HHT), occasionally thoracic aortopathy. | Plausible — not well quantified; arrhythmias rarely described, but fibrosis and remodeling suggested in mechanistic studies. | Myocardial fibrosis is noted in some cases; conduction remodeling described in experimental models. | Limited clinical arrhythmia data; arrhythmic risk likely underestimated due to focus on vascular/GI phenotypes. |
| ***SPARC*** | Osteonectin (SPARC) | Matricellular regulator essential for collagen deposition; promotes myocardial stiffening when overexpressed | 5q33.1 | Matricellular ECM remodeling / collagen deposition | Not classic vascular syndrome (rare OI type 17 association outside scope) | Emerging — fibrosis-driven conduction heterogeneity and arrhythmia susceptibility | Fibrosis signal on CMR; potential elevation of fibrosis biomarkers when active | SPARC remains an investigational fibrosis pathway; no SPARC-directed therapy or surveillance strategy has been established. |
| ***TGFB2*** | Transforming growth factor beta-2 | Ligand in the TGF-β pathway; regulates vascular development and remodeling | 1q41 | TGF-β / SMAD signaling | Loeys–Dietz syndrome type 4 (aortic aneurysm/dissection) | Atrial fibrillation, ventricular ectopy (plausible; reported in HTAD cohorts but not well quantified) | Aortic dilation, MVP/MAD occasionally present; fibrosis suspected but under-studied | Rhythm risk often underestimated; may occur independent of severe valve disease |
| ***TGFB3*** | Transforming growth factor beta-3 | TGF-β family cytokine; modulates ECM and cellular proliferation | 14q24.3 | TGF-β / SMAD signaling | Loeys–Dietz syndrome type 5 (aortic aneurysm/dissection, valvular disease) | AF, VT, arrhythmia in LDS5 (plausible–emerging); overlap with MVP/MAD | MVP/MAD geometry, basal fibrosis (reported in subsets) | Imaging markers (MAD) may be better predictors than aortic size; limited arrhythmia-focused studies |
| ***TGFBR1*** | Transforming growth factor-beta receptor type I | Serine/threonine kinase receptor for TGF-β; initiates SMAD signaling cascade | 9q22.33 | TGF-β / SMAD signaling | Loeys–Dietz syndrome type 1 (aggressive TAAD, arterial tortuosity) | AF, nonsustained VT (plausible; small cohorts, case reports) | Aortic aneurysm, valve disease, diffuse fibrosis in subsets | High surgical burden confounds rhythm phenotyping; postoperative AF frequent |
| ***TGFBR2*** | Transforming growth factor-beta receptor type II | Serine/threonine kinase that binds TGF-β ligands and activates TGFBR1 | 3p24.1 | TGF-β / SMAD signaling | Loeys–Dietz syndrome type 2 (aggressive TAAD, arterial aneurysms/dissections) | AF, ectopy, occasional VT (plausible; often with MVP/MAD or atrial enlargement) | MVP/MAD, atrial enlargement, fibrosis suspected | Rhythm issues sometimes dismissed as secondary to valvular disease; need dedicated surveillance |
| ***THSD4 (ADAMTSL6)*** | ADAMTS-like 6 (secreted microfibril-associated protein) | Promotes fibrillin-1 assembly; stabilizes microfibrils | 15q23 | Microfibrils / valve–annulus stabilization | Familial thoracic aortic aneurysm predisposition | Emerging — annular instability/MVP-MAD geometry and fibrosis provide VA substrate | MAD/MVP with basal LV fibrosis and Purkinje-triggered ectopy | Risk-stratify with MAD quantification + fibrosis imaging |
| ***TNS1*** | Tensin 1 | Focal-adhesion adaptor linking β-integrins to actin; traction-sensing at leaflet–annulus interface | 2q35 | Focal adhesion / integrin mechanotransduction | Human MVP locus (GWAS); myxomatous changes in mice; AV valve regurgitation in zebrafish | Emerging — impaired traction sensing → annular hypermobility, MAD, basal LV fibrosis, and Purkinje irritability typical of arrhythmogenic MVP | MAD quantification; basal fibrosis on CMR in higher-risk MVP | Incorporate MAD severity into risk stratification; monitor ventricular ectopy burden |
| ***TNXB*** | Tenascin-X | Organizes collagen fibrils; maintains interstitial ECM architecture | 6p21.33 | Proteoglycans / linkeropathies | Classical-like EDS; connective-tissue laxity ± vascular features | Plausible — annular destabilization and fibrosis amplify substrate susceptibility | Myxomatous valve/annular changes; diffuse fibrosis | Tissue fragility considerations for ablation/ICD |
| ***VCL*** | Vinculin | Focal-adhesion/costamere adaptor linking integrins to actin; stabilizes force transmission | 10q22.2 | Focal adhesion / mechanotransduction | Not a vascular syndrome; cardiomyocyte models show DCM with conduction abnormalities | Emerging — conduction defects and arrhythmic death in models; plausible modifier in CTDs | Fibrosis and conduction disease (bundle/PR/QRS abnormalities) may precede systolic dysfunction | Emphasize rhythm monitoring where mechanical load is abnormal (e.g., MVP/MAD CTDs) |

This table summarizes genes and molecular pathways that may influence arrhythmia susceptibility through extracellular matrix remodeling, valve and annular abnormalities, mechanotransduction, fibrosis, and downstream electrical remodeling. Evidence levels reflect the strength of the available human and experimental literature. Established indicates a reproducible human association with the relevant structural or arrhythmic phenotype; Plausible indicates supportive human or experimental evidence without a validated direct association; and Emerging indicates predominantly mechanistic, preclinical, or limited clinical evidence. These categories do not establish gene-specific arrhythmic causality or provide genotype-specific risk estimates. Clinical considerations are intended to place the biologic evidence in context and should not be interpreted as formal screening, surveillance, or treatment recommendations.

Abbreviations: AF = atrial fibrillation; VA = ventricular arrhythmias; VT = ventricular tachycardia; VF = ventricular fibrillation; PVC(s) = premature ventricular contraction(s); SCD = sudden cardiac death; ICD = implantable cardioverter-defibrillator; PR = PR interval; QRS = QRS duration; CMR = cardiac magnetic resonance; LGE = late gadolinium enhancement; LV = left ventricle/left ventricular; MR = mitral regurgitation; MVP = mitral valve prolapse; MAD = mitral annular disjunction; BAV = bicuspid aortic valve; CTD(s) = connective tissue disease(s); EDS = Ehlers–Danlos syndrome; MFS = Marfan syndrome; LDS = Loeys–Dietz syndrome; TAAD = thoracic aortic aneurysm/dissection; HTAD = heritable thoracic aortic disease; PDA = patent ductus arteriosus; JPS-HHT = juvenile polyposis syndrome–hereditary hemorrhagic telangiectasia; DCM = dilated cardiomyopathy; OI = osteogenesis imperfecta; SMC = smooth muscle cell; VSMC = vascular smooth muscle cell; VIC = valve interstitial cell; ECM = extracellular matrix; GAG(s) = glycosaminoglycan(s); SLRP = small leucine-rich proteoglycan; AR = autosomal recessive (as in “AR cutis laxa”); AV = atrioventricular (as in “AV valve”); EF = ejection fraction; GWAS = genome-wide association study; ± = with or without.

Table 2. Clinical Studies Reporting Arrhythmic Phenotypes and Related Structural Findings in Connective Tissue Disorders

| **Disorder** | **Study population and design** | **Cohort size** | **Reported arrhythmic findings** | **Structural or imaging findings** | **Principal limitations** |
| --- | --- | --- | --- | --- | --- |
| **Genetically confirmed HTAD** | International multicenter retrospective cohort of patients with pathogenic or likely pathogenic variants in *FBN1*, TGF-β signaling genes, or *ACTA2* | 3,219 screened; 101 with non-aortic cardiac disease | Non-aortic cardiac disease was identified in 3.1% of screened patients. Among 142 reported manifestations, 47 (33%) were AF or AFL and 27 (19%) were VT, VF, or SCD. | Prior cardiovascular surgery was present in 80% and severe valvular disease in 58%; 18% developed non-aortic cardiac disease without either. | Rhythm surveillance was not standardized. The percentages for AF/AFL and VT/VF/SCD describe the distribution of reported manifestations rather than prevalence among all patients with HTAD. |
| **MFS, LDS, and vEDS** | Retrospective comparative cohort; formally published conference abstract | 1,032 | AF or AFL was documented in 23.8% of patients with MFS, 30.9% with LDS, and 11.4% with vEDS. | Not reported in the published abstract. | Abstract-level reporting with limited methodological detail; selected clinical populations without a general-population comparator. |
| **MFS** | Nationwide retrospective analysis of hospitalizations in the National Inpatient Sample from 2005 through 2014 | 12,079 hospitalizations | An arrhythmia was coded in 1,893 hospitalizations (15.7%). A co-diagnosis of POTS was present in 4.9%. VT was associated with the highest in-hospital mortality among the rhythm categories examined. | Structural and imaging findings were not available. | Hospitalization-level administrative data based on ICD-9-CM coding; repeat hospitalizations could not be distinguished from unique patients, and the findings should not be interpreted as population-level prevalence. |
| **MFS** | Retrospective cohort of genetically confirmed MFS with echocardiography and ambulatory rhythm assessment | 142 | Ventricular ectopy and nonsustained VT were more frequent in patients with MAD. Sustained VT or SCD occurred exclusively among patients with MAD. | MAD was present in approximately one-third of patients and was closely associated with MVP. More extensive MAD was also associated with mitral valve intervention and aortic events. | Single referral cohort with few major arrhythmic events; MAD was assessed using the imaging definition applied at the time of the study. |
| **MFS** | Cross-sectional, single-center CMR study of patients with a pathogenic or likely pathogenic *FBN1* variant | 32 | Twenty-four-hour Holter data were available in 28 patients, but the study was not designed to establish arrhythmia prevalence or prospective arrhythmic risk. | Diffuse myocardial fibrosis was identified in 6 patients (21%); focal fibrosis was absent. MAD was associated with larger ventricular volumes and lower ventricular function. | Small, cross-sectional imaging cohort without a control group or longitudinal arrhythmic outcomes. |
| **MFS** | Multicenter retrospective study of patients undergoing catheter ablation for AF or AFL | 67 underwent ablation; 41 underwent rhythm-targeted AF ablation | Among patients undergoing AF ablation, 34 of 41 (82.9%) experienced recurrent atrial arrhythmia and 20 (48.8%) underwent multiple procedures. Complications occurred in 19 of 67 patients (28.4%) undergoing ablation. | Echocardiographic characteristics were collected, but imaging predictors were not the primary focus. | Highly selected procedural cohort without a non-MFS control group; ablation indications and procedural approaches were heterogeneous. |
| **MFS versus vEDS** | Propensity-matched international administrative-database analysis; formally published conference abstract | 1,091 patients per matched group | VT was documented in 2.6% of patients with MFS and 0.9% of patients with vEDS. | Structural and imaging findings were not available. | Reliance on diagnostic coding without manual rhythm adjudication; residual confounding and limited methodological detail in the published abstract. |
| **LDS** | Multicenter retrospective cohort evaluating atrial, ventricular, and conduction abnormalities | 94 | Atrial arrhythmias occurred in 31.9%, including AF in 28.7%, AFL in 12.7%, and SVT in 19.1%. Ventricular arrhythmias occurred in 20.2%, and conduction abnormalities occurred in 37.2%. | MVP and MAD were present in 35.1% and 11.7%, respectively, and were each associated with ventricular arrhythmias. | Referral cohort with a high burden of cardiovascular surgery; rhythm ascertainment depended on available clinical testing and documentation. |
| **LDS** | Multicenter retrospective analysis of postoperative outcomes and valvular involvement | 94 | New-onset postoperative arrhythmias occurred in more than 40% of patients, predominantly AF or AFL. | Mitral and tricuspid valve disease were common. Mechanical valve recipients experienced substantial anticoagulation-associated bleeding. | Surgically enriched population; postoperative findings should not be interpreted as arrhythmia prevalence in unselected patients with LDS. |
| **LDS** | Retrospective CMR cohort of adults undergoing routine aortic imaging | 46 | Sustained VT occurred only in the MAD group, although the number of severe arrhythmic events was small. | MAD was present in 17 patients (37%) and was associated with younger age at aortic events and progressive aortic root enlargement. | Small, CMR-selected cohort with few clinical events and limited power to define arrhythmic risk. |
| **EDS and HSD** | Retrospective study of symptomatic patients evaluated with an implantable loop recorder | 116 evaluated; 31 received an implantable loop recorder | Among monitored patients, symptomatic sinus tachycardia occurred in 51.6%, PVCs in 25.8%, SVT in 19.4%, and VT in 3.2%. Findings prompted PVC ablation in one patient and ICD implantation in one patient with symptomatic VT. | Structural imaging findings were not a primary study outcome. | Symptom-selected population composed predominantly of women with hypermobile EDS or HSD; findings are not generalizable to all EDS subtypes or asymptomatic patients. |
| **Williams syndrome** | Retrospective pediatric cohort undergoing ambulatory ECG monitoring | 74 | Arrhythmias were identified in 9 patients (12%). Atrial tachycardia was the most common arrhythmia, occurring in 6 patients (8% of the total cohort). | Arrhythmias were not associated with the severity of structural heart disease. | Pediatric tertiary-care cohort selected for ambulatory monitoring; small number of arrhythmic events. |
| **Osteogenesis imperfecta** | Systematic review of case-control studies and cohort series | 22 studies | The only population-based study addressing atrial arrhythmias reported a higher risk of AF or AFL in osteogenesis imperfecta than in matched controls, with a subhazard ratio of 1.7. | Valvular disease and larger aortic root dimensions were reported more frequently in patients with osteogenesis imperfecta than in controls. | AF and AFL were evaluated in only one included study. Included studies were heterogeneous in age, osteogenesis imperfecta subtype, cardiovascular assessment, and design. |

Data are presented as reported by the individual studies. Because the included populations differed in age, referral setting, surgical history, monitoring intensity, imaging methods, and outcome definitions, estimates should not be compared directly or interpreted as population-level prevalence. Percentages reported among patients with identified non-aortic cardiac disease, patients undergoing ablation, or symptomatic patients receiving an implantable loop recorder do not represent prevalence within the broader disorder. Hospitalization-based estimates may include repeated admissions by the same patient. The comparative MFS, LDS, and vEDS cohort and the MFS-versus-vEDS ventricular tachycardia analysis were formally published conference abstracts. POTS was a co-primary outcome in the nationwide MFS study but is an autonomic disorder rather than a primary cardiac arrhythmia.

Sources: Genetically confirmed HTAD cohort, reference 25; comparative MFS, LDS, and vEDS cohort, reference 101; nationwide MFS inpatient study, reference 106; MFS MAD cohort, reference 69; MFS CMR fibrosis cohort, reference 107; MFS ablation cohort, reference 120; MFS-versus-vEDS ventricular tachycardia analysis, reference 102; comprehensive LDS arrhythmia cohort, reference 110; LDS postoperative cohort, reference 104; LDS CMR cohort, reference 111; EDS and HSD loop-recorder cohort, reference 114; Williams syndrome cohort, reference 115; and osteogenesis imperfecta systematic review, reference 116.

Abbreviations: AF, atrial fibrillation; AFL, atrial flutter; CMR, cardiac magnetic resonance; ECG, electrocardiography; EDS, Ehlers-Danlos syndrome; HSD, hypermobility spectrum disorder; HTAD, heritable thoracic aortic disease; ICD, implantable cardioverter-defibrillator; LDS, Loeys-Dietz syndrome; MAD, mitral annular disjunction; MFS, Marfan syndrome; MVP, mitral valve prolapse; POTS, postural orthostatic tachycardia syndrome; PVC, premature ventricular contraction; SCD, sudden cardiac death; SVT, supraventricular tachycardia; vEDS, vascular Ehlers-Danlos syndrome; VF, ventricular fibrillation; VT, ventricular tachycardia.
